# Implementing a male‐specific ART counselling curriculum: a quality assessment with healthcare workers in Malawi

**DOI:** 10.1002/jia2.26270

**Published:** 2024-07-22

**Authors:** Isabella Robson, Misheck Mphande, Jiyoung Lee, Julie Anne Hubbard, Joseph Daniels, Khumbo Phiri, Elijah Chikuse, Thomas J. Coates, Morna Cornell, Kathryn Dovel

**Affiliations:** ^1^ Division of Infectious Diseases David Geffen School of Medicine University of California Los Angeles Los Angeles California USA; ^2^ Implementation Science Department Partners in Hope Lilongwe Malawi; ^3^ David Geffen School of Medicine University of California Los Angeles Los Angeles California USA; ^4^ Edson College of Nursing and Health Innovation Arizona State University Phoenix Arizona USA; ^5^ University of California Global Health Institute San Francisco California USA; ^6^ Centre for Infectious Disease Epidemiology & Research School of Public Health University of Cape Town Cape Town South Africa

**Keywords:** ART, healthcare workers, HIV counselling, Malawi, male‐specific, men

## Abstract

**Introduction:**

There is little HIV counselling that directly meets the needs of men in Eastern and Southern Africa, limiting men's knowledge about the benefits of HIV treatment and how to overcome barriers to engagement, contributing to poorer HIV‐related outcomes than women. Male‐specific approaches are needed to improve men's outcomes but may be difficult for healthcare workers (HCWs) to implement with fidelity and quality in low‐resource settings. We developed a male‐specific counselling curriculum which was implemented by male HCWs and then conducted a mixed‐methods quality assessment.

**Methods:**

We audio‐recorded counselling sessions to assess the quality of implementation (*n* = 50) by male HCWs from two cadres (nurse, *n* = 10 and lay cadre, *n* = 10) and conducted focus group discussions (FGDs) with HCWs at 6 and 9 months after rollout to understand barriers and facilitators to implementation. Counselling sessions and FGDs were translated, transcribed and analysed using thematic analysis adapted from WHO Quality Counselling Guidelines. We assessed if sessions were respectful, informative, interactive, motivating and included tailored action plans for overcoming barriers to care. All data were collected September 2021−June 2022.

**Results:**

All sessions used respectful, non‐judgemental language. Sessions were highly interactive with most HCWs frequently asking open‐ended questions (*n* = 46, 92%) and often incorporating motivational explanations of how antiretroviral therapy contributes to life goals (*n* = 42, 84%). Few sessions included individually tailored action plans for clients to overcome barriers to care (*n* = 9, 18%). New counselling themes were well covered; however, occasionally themes of self‐compassion and safe sex were not covered during sessions (*n* = 16 and *n* = 11). HCWs believed that having male HCWs conduct counselling, ongoing professional development and keeping detailed counselling notes facilitated quality implementation. Perceived barriers included curriculum length and client hesitancy to participate in action plan development. Findings were similar across cadres.

**Conclusions:**

Implementing high‐quality male‐specific counselling using male nurses and/or lay cadre is feasible. Efforts to utilize lay cadres should be prioritized, particularly in low‐resource settings. Programmes should provide comprehensive job aids to support HCWs. Ongoing training and professional development are needed to (1) improve HCWs’ skills in tailored action plans, and (2) sensitize HCWs to the need for self‐compassion within male clients to promote holistic sexual health.

## 1 INTRODUCTION

Men in Eastern and Southern Africa have poorer HIV‐related outcomes than women [1, 2, 3]. Men are less likely to be tested for HIV, retained in HIV care and reach viral suppression than women [2, 4–6]. HIV services in Malawi, and Eastern and Southern Africa broadly, have largely focused on women and children with few services tailored to men [7, 8].

Intersecting factors negatively influence men's engagement with HIV care. In several Eastern and Southern African contexts, including Malawi and Tanzania, negative health service experiences, such as long wait‐times, lack of privacy, inflexible services and services tailored to women, as well as lack of empathy from healthcare workers (HCWs) discourage men's use of services [9, 10, 11, 12]. In turn, men in these settings do not gain up‐to‐date knowledge on HIV treatment and its benefits, like Undetectable = Untransmissible (U = U) and limited side effects [13, 14]. Work schedules that conflict with facility services and frequent‐related travel deprioritize care engagement [10, 15]. Further, men find it difficult to navigate social expectations of manhood and anticipated stigma to engage in treatment [5, 16, 17]. Without services that embrace these factors and address men's knowledge gaps, men will continue to have poor retention, high levels of attrition and cyclical disengagement in Eastern and Southern Africa [18, 19]. There is an urgent need for programmes to quickly re‐engage men experiencing treatment interruption, in part due to inadequate HIV counselling and care, and to adjust the course for men by improving the HIV treatment counselling that is provided to them.

Person‐centred counselling may help address gaps in men's HIV knowledge, motivations for treatment engagement and ability to connect with HCWs. Person‐centred counselling offers a solution‐focused approach in which counselling is tailored to the unique, immediate needs of the individual, and counsellors address personal barriers to develop solutions together with the client [20, 21]. It situates client and counsellor as equal partners in shared health‐related decision‐making [22, 23]. For clients throughout the region, person‐centred counselling has been shown to improve health‐related literacy in Uganda and Ethiopia [24, 25, 26], demonstrate how the benefits of lifelong treatment can align with life goals [27, 28] and improve HIV treatment outcomes in South Africa [21, 29]. This approach could be critical in improving antiretroviral therapy (ART) initiation and sustained retention for men [5, 30]. Crucially for this population, person‐centred counselling aims to demonstrate that the value of treatment engagement outweighs its perceived costs [31], something men may struggle to balance particularly when needing to prioritize income generation activities [9, 32]. Unlike common lecture‐based approaches to HIV counselling, person‐centred counselling may appeal to men because it fosters autonomy and care self‐management by leveraging HIV knowledge and skill building [33, 34, 35].

However, person‐centred counselling can be difficult to implement with fidelity in routine settings. Offering tailored messaging to specific populations and individuals, a critical component of person‐centred counselling, may not be feasible for busy HCWs without significant oversight that is also often not possible [36]. HCWs in resource‐limited settings like Malawi often have overwhelming workloads [37, 38], limited training [39] and limited time available to implement and improve skills in person‐centred counselling, receiving limited monitoring and evaluation to promote person‐centred counselling quality and fidelity [40, 41]. There may be additional barriers to person‐centred counselling for men specifically. HCWs have perceived men as difficult and stubborn clients responsible for their own experiences of treatment interruption [42, 43] which can limit HCW buy‐in to tailored interventions and may negatively impact the provision of high‐quality, person‐centred counselling for male clients. Reciprocal poor experiences between men and HCWs [44] mean there is a lack of established patient‐provider communication for men that may otherwise help to improve experiences of HIV services.

To address implementation barriers, we developed a male‐specific (person‐centred) counselling curriculum for men. We then conducted a mixed‐methods study to assess the quality and fidelity of its implementation within designated facilities in Malawi and assess buy‐in among implementing HCWs.

## 2 METHODS

### 2.1 Setting

This mixed methods sub‐study was embedded within two parent trials: Engaging Men Through Differentiated Care to Improve ART Initiation and Viral Suppression Among Men in Malawi (ENGAGE) (clinicaltrials.gov NCT04858243) and Identifying Efficient Linkage Strategies for Men (IDEaL) (clinicaltrials.gov NCT05137210). ENGAGE and IDEaL are individually randomized trials that test the impact of varying interventions on ART (re‐)initiation and 6‐month retention and/or viral suppression among men in Malawi. In total, 24 health facilities across Malawi participated in the trials, 20 of which were included in our study, and 1303 men were enrolled (734 in ENGAGE and 569 in IDEaL). Interventions included male‐specific counselling offered alongside: facility‐based (re)initiation, one‐time home‐based (re)initiation, home‐based (re)initiation and dispensing for 3 months, and stepped interventions including motivational interviewing. Additional details regarding the trials can be found elsewhere [45, 46]. All data for this sub‐study were collected between September 2021 and June 2022. The study was approved by the National Human Sciences Research Committee of Malawi (#20/07/2562).

### 2.2 Male‐specific counselling intervention

#### 2.2.1 Curriculum

All intervention arms within ENGAGE and IDEaL included male‐specific counselling. Methods for developing and piloting the male‐centred counselling curriculum are described in detail elsewhere [47]. Briefly, we adapted the Malawi Ministry of Health (MoH) standard HIV Testing and Counselling curriculum to include key topics identified as important to men in Malawi, based on prior literature and formative qualitative research with men and HCWs [10, 43, 48, 49, 50, 51, 52]. The curriculum aligned with person‐centred counselling and motivational interviewing principles [53, 54], whereby counselling was meant to facilitate identifying how treatment can contribute to men's priorities/goals, identifying challenges to treatment success and developing tailored strategies to overcome challenges, in order to facilitate highly interactive counselling sessions tailored to clients’ individual needs [21].

**Figure 1:**
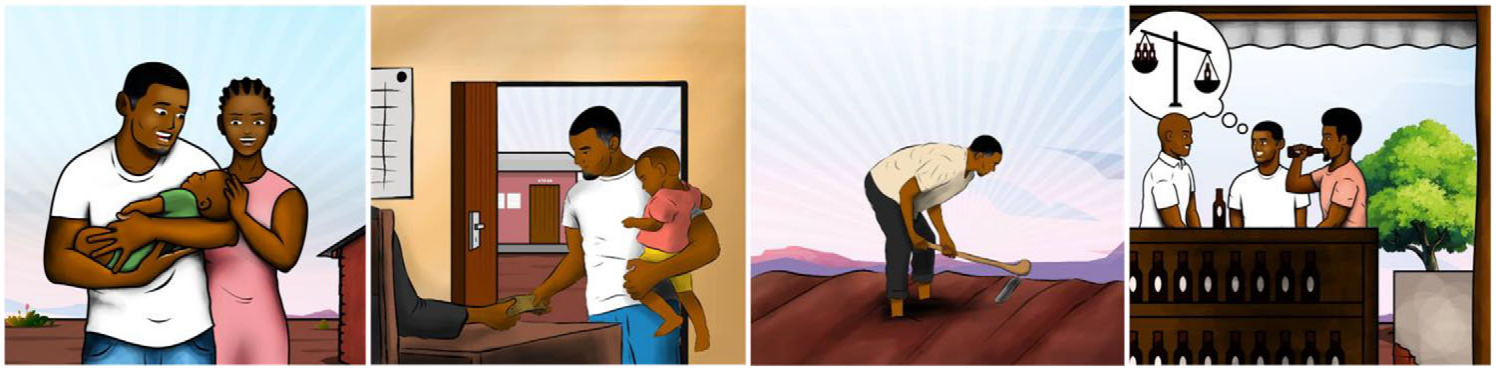
Examples of male‐specific antiretroviral therapy (ART) counselling curriculum graphics depicting men as family providers, working and engaging in common behaviour such as drinking alcohol.

The final curriculum consisted of 17 topics, including four new topics not previously included in MoH counselling curriculum and three MoH topics that were modified to meet the unique needs and priorities of men (Table 1). Key messaging for men focused on (1) the benefits of ART adherence for enhancing health, (2) the relationship between ART adherence and men's ability to provide for their families and maintain their roles within the community, and (3) the importance of self‐compassion for lifelong adherence and facility attendance, emphasizing that life is complicated, and men should not feel undue guilt if they miss a dose. The messaging was intended to be empowering and help men explore how treatment adherence supports rather than conflicts with their personal goals and priorities. A small flipchart with the counselling curriculum was developed and included a job aid for HCWs (reminding them about key points within each topic) and graphics to help clients connect with the material, all designed by a local artist and tailored to men and the local context (Supplementary Appendix A). Images featured a central male character who was intended to be a relatable role model figure to most men in rural and semi‐urban Malawi. The character was depicted in various scenarios that were identified as motivating from the formative work (see Figure 1) and images were visible to the men during counselling sessions. HCWs were able to ask men what they saw and if it related to their own life. Only male HCWs were recruited so that they could relate to these goals and engage with clients as men [55, 56].

**Table 1:** New and modified male‐specific counselling themes included in the male‐specific counselling curriculum [47]

Theme	Male‐specific modification
New themes	
How treatment contributes to men's goals	We work with men to frame their possible future goals based on their health. Prior to this, men argued that antiretroviral therapy (ART) engagement competed with business and agriculture priorities. We discuss how HIV services contribute to their key goals. We employ motivational interviewing skills to discussing ART as an integral component to achieve goals.
Feeling healthy on treatment and low ART knowledge	We explicitly acknowledge challenges of taking ART when feeling healthy and ask men to reflect on their own experience. We discuss how taking ART while healthy will prevent disruption of earning prospects and support strong business and better families, using local analogies and graphics that resonate with Malawian men.
Navigating health system	We discuss barriers men have experienced when seeking health services at facilities and discuss how to overcome them, and notably, to report poor services. We address the pre‐existing discord between healthcare workers (HCWs) and men.
Self‐compassion/patience for lifelong treatment	We normalize the fact that men may forget a dose, feel guilty and panic. We highlight the importance of returning to care as soon as possible. We discuss alcohol use and fears about long‐term treatment adherence. We acknowledge individual concerns about competing responsibilities, fear of disclosure and treatment fatigue, and stress that such fears are normal.

Modified themes	
Status disclosure to male friends/family	Beyond disclosure to their sexual partners, we highlight the importance of disclosing to their male social support system. We discuss who is important to them, how to disclose to other men and how to get social support for ART disclosure.
Treatment as prevention	In addition to the general treatment as prevention message, we provide a detailed description about how treatment as prevention works, men's role in preventing vertical transmission, and how these concepts contribute to men's role as provider and building a stronger future.
Understanding ART (side effects)	Ministry of Health (MOH) counselling simply describes potential side effects and tells clients to report to clinic if they persist. We focus on the changes in ART regimen including that the new dolutegravir regimen has fewer side effects than the older regimens.

#### 2.2.2 Training

Two cadres of male HCWs implemented the male‐specific counselling curriculum: a lay cadre of community HCWs, named Treatment Supporters in Malawi, responsible for HIV‐related tracing activities and HIV‐related counselling; and nurses responsible for delivering HIV counselling and treatment services both in facility and community settings. HCWs initially received a 2.5‐day training that included: (1) sensitization to the challenges and needs of men as ART clients (.5 days); (2) best practices for implementing person‐centred counselling with motivational interviewing principles (.5 days); (3) review of curriculum (.5 days); and (4) peer role play and feedback on counselling strategies (1 day). HCWs were employed specifically for the studies but did not receive any incentives for providing male‐specific counselling. After training and curriculum rollout, HCWs met bi‐weekly as a group with their supervisor to discuss challenges and facilitators to curriculum implementation.

#### 2.2.3 Implementation

Male‐specific counselling took place one‐on‐one in the community (at home or the surrounding area, as preferred by participants). During study enrolment, participants confirmed their home address and indicated preferred locations to be traced by HCWs. Tracing was conducted by phone and in person and took place up to three times before a participant was considered not found (if all attempts were unsuccessful). Counselling was conducted in the local language (Chichewa) using the visual job aid and lasted on average 40−70 minutes. Clients received one to four counselling sessions, depending on the study arm and need for additional counselling based on clients’ desire for additional counselling and/or clients’ engagement with HIV treatment services.

### 2.3 Data collection

#### 2.3.1 Counselling sessions

Between September and November 2021, HCWs requested consent to record all counselling sessions. We recorded a total of 160 male‐specific counselling sessions from 18 male HCWs (nine nurses, nine lay cadre). We randomly selected 50 counselling sessions for analysis and stratified the sample by cadre (lay cadre and nurse), with a mean of three counselling sessions per HCW. For clients who received multiple counselling sessions, we selected their first session to understand the full scope of topics covered as follow‐up counselling sessions focused only on recurring barriers to men's care and may not have reflected the full curriculum. Selected sessions were transcribed, translated into English and reviewed against the recording for quality assurance.

#### 2.3.2 Focus group discussions with HCWs

We conducted focus group discussions (FGDs) with the male HCWs who implemented the male‐specific counselling at 6 and 9 months after counselling rollout with questions developed based on intervention design, counselling topics and prompts for self‐reflection to assess buy‐in, longitudinal perceptions of curriculum, and barriers and facilitators to implementing the curriculum with fidelity. FGDs were conducted separately for nurses (*n* = 9) and lay cadre (*n* = 9), totalling two FGDs per cadre. All HCWs were brought together at a central location so that FGDs could be conducted in person. FGDs were conducted by trained facilitators in a combination of local language and English, based on participant preferences, and were recorded, transcribed, and translated into English. Transcripts were reviewed against the recording for quality assurance.

### 2.4 Measuring fidelity and quality in counselling sessions

We analysed counselling data with a focus on two components: fidelity and quality. We defined fidelity as HCWs covering all topics included in the counselling curriculum job aid during the first counselling sessions. We measured if HCWs covered each of the 17 curriculum topics by including counts of the number of topics covered during each counselling session (defined as covered, or not covered at all).

We defined quality as adhering to best practices of person‐centred counselling, as covered in the training. We measured the quality of counselling sessions according to the World Health Organization (WHO) building blocks for quality counselling [57], adapted to the local context and used for routine programme monitoring in Malawi. We included seven of the eight WHO core domains for quality counselling. We added “Motivate,” one additional domain that was central to curriculum development [47] and to men's use of HIV services in Malawi, providing male‐specific, motivational explanations for the use of HIV services (Table 2).

**Table 2:** Key building blocks of quality person‐centred counselling for men

Building block	Definition	Example script
**Setting** Private, comfortable environment	Counselling is conducted in a private environment chosen by the clients, to enable them to speak freely without fear of unwanted disclosure.	*NA—no script needed if counselling in a private area*
**Rapport** Build a relationship with client	Healthcare worker (HCW) builds a relationship with the client by asking him to share his story.	*What problems have you encountered in the past in taking your medication? Tell me more about what happened exactly*. *Use non‐verbal cues to build rapport*.
**Respect** Speak in a non‐judgemental, empathetic way	HCW uses empathetic language towards client that does not blame or shame him for his status or experiences.	*Don't feel bad that you have struggled to remember to take your medication every day. We all struggle, it's natural*.
**Open** Open‐ended questions	HCW asks open‐ended questions to elicit further information about client's knowledge and experience with HIV and treatment.	*When you went travelling, what did you do to ensure you could continue taking medication?*
**Respond** Respond to client contributions	HCW follows up on client's responses to better understand the client's experiences.	*You mentioned that you are not married but you have a partner, does she know about your status? Have you thought about disclosing to her?*
**Inform** Respond to client questions	HCW answers client's questions accurately and in detail.	*NA—no script needed, HCWs should respond to specific questions raised by client during sessions*.
**Act** Work with client to make action steps for the future	HCW and client work together to develop a tangible action plan to overcome barriers faced.	*If you can't afford to travel to the clinic monthly, what other plan can you make to get your medication? Is there a neighbour with a bicycle you can borrow? Let's talk about how you can ask him*.
**Motivate** Provide male‐specific, motivational explanations for health education material	HCW describes *why* a topic is important rather than just stating that it is. Explanations can be tailored towards the specific client based on his story, or to men in Malawi generally if client's story is unknown.	*You shared with me that you are worried about feeding your children. Even if you feel healthy, you should still take ART because keeping healthy will mean you can feel fit and strong enough to work on your farm and provide food for them*.

For this analysis, we included six of the eight quality measures. We excluded measures “Setting” and “Rapport” because of the difficulty to objectively measure from recorded sessions. Measures “Open” and “Respond” were used to assess overall interactivity within discussions, characterized by back‐and‐forth exchanges and HCWs asking open‐ended questions and following up on responses.

Each component was rated on a scale of “frequent,” “sometimes,” “never” or “not applicable.” Definitions for each measure are described in Table 3. Sessions that included quality domains frequently were assessed to be of higher quality.

**Table 3:** Definition summaries for measures included in quality analysis of implementation of male‐specific counselling

	Respect	Open	Respond	Inform	Act	Motivate
**Frequent**	Non‐judgemental language used 100% of the time	≥1 open‐ended questions for >50% topics covered	≥1 follow‐up response for >50% of times client contributed	Detailed response to 100% of client's questions	Client and counsellor formulated tailored action plan together at any point during the session	≥1 tailored explanation for >50% topics covered
**Sometimes**	Non‐judgemental language used ≥50% of the time	≥1 open‐ended questions for ≤50% topics covered	≥1 follow‐up response for ≤50% of times client contributed	Detailed response to 50% of client's questions	N/A	≥1 tailored explanation for ≤50% topics covered
**Never**	Judgemental language ever used	No open‐ended questions in any topics covered	No follow‐up response made any time client contributed	No detailed response to any of client's questions	No tailored action plan ever made between client and counsellor	No tailored explanation for any topics covered
**Not applicable**	N/A	N/A	No personal information given by client to follow‐up	No questions asked by client	N/A	N/A

### 2.5 Data analysis

We analysed both counselling sessions and HCW FGDs in Atlas.ti v.9 using thematic analysis [58, 59]. We developed codes *a priori* based on existing literature [60], and the quality counselling building blocks and added additional codes as new themes emerged. We summarized codes and identified overarching themes. For counselling sessions, we also completed structured memos concurrently to coding to rate the incidence of each quality measure within each session and provide cross‐comparison between coders (JL and IR).

## 3 RESULTS

### 3.1 Demographics of HCWs

The mean HCW age was 31 years (IQR: 27−35), all were male (*n* = 18), and most had been providing HIV services for 2 or more years (11/18, 61%) prior to the study.

### 3.2 Fidelity

**Figure 2:**
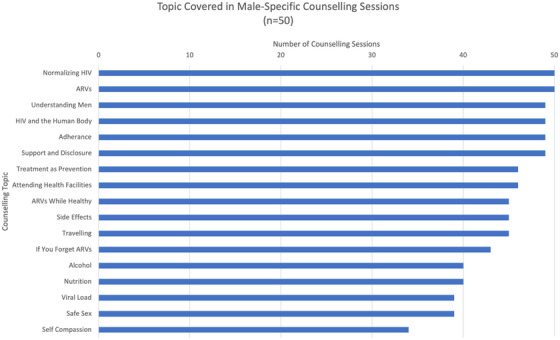
Topic coverage of male‐specific curriculum topics during counselling sessions (*n* = 50).

The majority of curriculum topics were covered in all counselling sessions, with an average of 15 of 17 (88%) topics covered in each session. The most frequently skipped topics were self‐compassion (skipped in 16/50 sessions [32%]) and safe sex, including condom use (skipped in 11/50 sessions [22%]) (Figure 2).

### 3.3 Quality

Counselling frequently had high levels of quality, creating open, respectful conversations and responding to client needs. All sessions included the domain “Respect”, using non‐judgemental language. Counselling sessions were highly interactive, characterized by both “Open” and “Respond” domains (Table 4). Most sessions (92%) frequently included open‐ended questions to clients; none of the sessions did *not* include open‐ended questions. In 94% of sessions, HCWs not only answered client questions but followed‐up at least once to solicit more detail or encourage sharing of personal experiences. For domain “Inform,” nearly all client questions (96%) were answered completely by HCWs. Overall, for domain “Motivate,” in 84% of sessions, HCWs used motivational explanations to describe how counselling topics directly applied to clients’ personal lives (i.e. why they should be important to the client).

**Table 4:** Quality measure results from 50 male‐specific counselling sessions conducted by 18 healthcare workers

	Respect	Open	Respond	Inform	Act	Motivate
*n* = 50	Accepting/non‐judgemental language used towards client *n* (%)	Open‐ended questions asked by counsellor *n* (%)	Follow‐up engagement made by counsellor *n* (%)	Client questions answered in detail by counsellor *n* (%)	Action plan developed *n* (%)	Motivational explanations provided by counsellor *n* (%)
**Frequent**	50 (100)	46 (92)	25 (50)	27 (96)	9 (18)	6 (12)
**Sometimes**	0 (0)	4 (8)	22 (44)	0 (0)	N/A	36 (72)
**Never**	0 (0)	0 (0)	3 (6)	1 (2)	41 (82)	8 (16)
**N/A**	N/A	N/A	0 (0)	22 (44)	N/A	N/A

A key domain of the WHO guidelines was “Act,” whereby HCW and client co‐develop a clear action plan based on clients’ personal experiences and barriers to care. This domain was poorly implemented with only 18% of sessions (9/50) including clear action plans. When action plans were formulated, they focused on strategies for adherence and disclosure to partners or relatives; one session included a plan to manage transportation costs for facility visits. Findings were similar across nurses and lay cadre (Supplementary Appendix B).

### 3.4 Quality by topic

**Figure 3:**
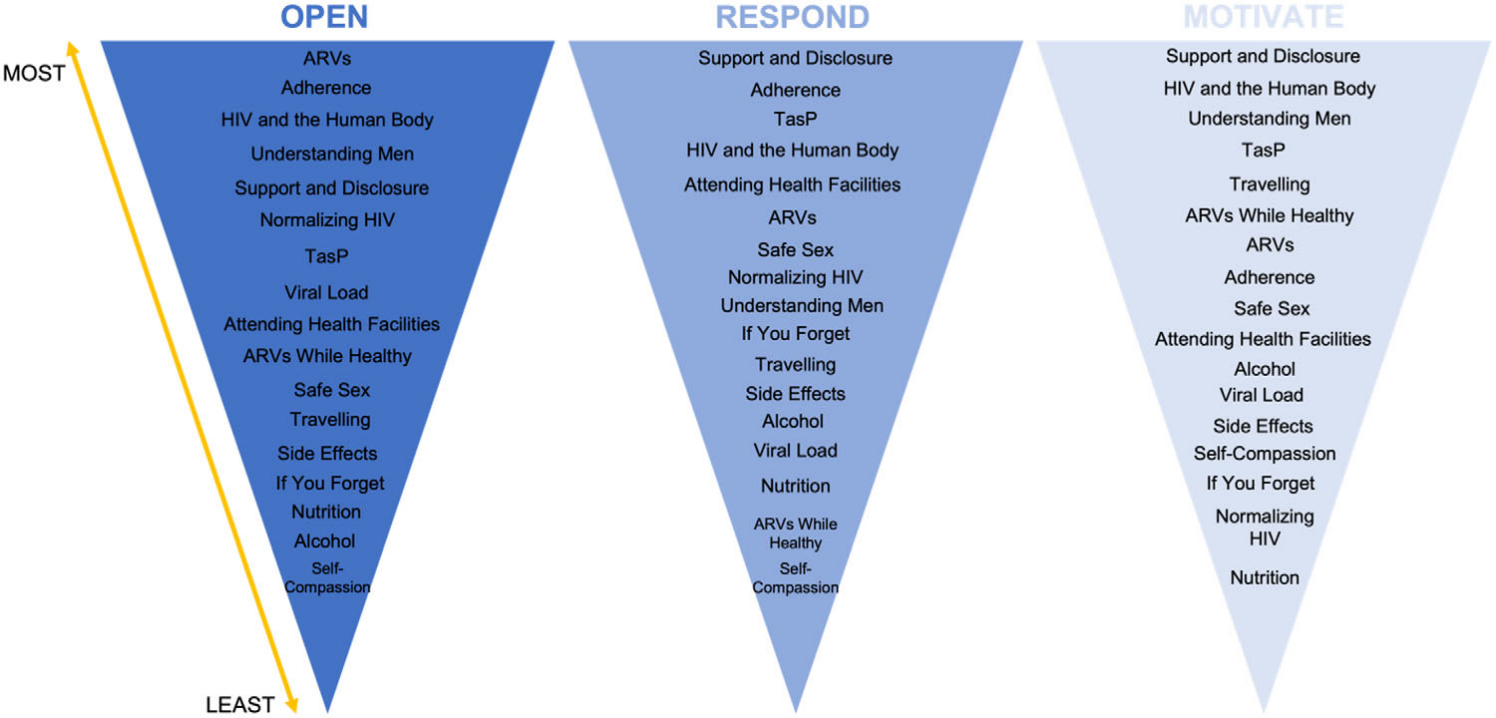
Quality per topic of measures Open, Respond and Motivate during 50 male‐specific counselling sessions.

We examined the extent to which counselling topics included quality domains, focusing on how well individual topics incorporated the components “Motivate,” “Open” and “Respond” (Figure 3). The topics “Social Support and Disclosure,” “HIV and the Human Body,” and “Understanding Men” were most likely to be highly interactive and include motivational language that was tailored to men's needs (“Motivate,” “Open” and “Respond” components included in ≥75% of these topics). The topics “Self‐Compassion,” “Nutrition,” “Alcohol” and “Side Effects” were rarely interactive and lacked motivational language—these topics largely used didactic counselling strategies that do not align with person‐centred care.

Men most frequently asked questions on the topics of “Attending Health Facilities,” “If You Forget ARVs,” “ARVs,” “Side Effects,” “Adherence” and “Travelling,” suggesting that these topics are priorities for men (Table 5).

**Table 5:** Topics of questions asked by men during male‐specific counselling sessions and proportion of questions asked for each topic (*n* = 49 questions)

Topic	Number and proportion of questions asked *n* (%)
If You Forget ARVs: What to do if you forget to take ARVs each day?	9 (19%)
Attending Health Facilities: What to do/where to go/who to talk to when you go to the facility for an appointment?	9 (19%)
ARVs: How do you take ARVs?	6 (12%)
Side Effects: What side effects do ARVs have?	4 (8%)
Adherence: How to be adherent to ARVs?	4 (8%)
Travelling: How do you remain adherent to ARVs while travelling?	4 (8%)
Treatment as Prevention: How does taking ARVs protect others?	3 (6%)
Support and Disclosure: Who to disclose to and how to disclose to gain support?	3 (6%)
Alcohol: Can you take ARVs and drink alcohol?	2 (4%)
Nutrition: How can good nutrition support adherence?	2 (4%)
Normalizing HIV: Who has HIV in the population?	1 (2%)
Viral Load: What is your viral load and when/how is it measured?	1 (2%)
Safe Sex: How can you have safe sex?	1 (2%)

### 3.5 Provider perceptions: FGDs

HCWs demonstrated high levels of buy‐in to the new curriculum and generally believed they were implementing high‐quality male‐specific counselling with fidelity. They liked the curriculum and believed the person‐centred style allowed them to connect with male clients more effectively through interactive sessions that encouraged clients to share opinions and ask questions as equal participants. HCWs believed that promoting equity within interactions meant that HCWs could build strong rapports with clients, allowing clients to guide the counselling session based on their personal experiences and barriers to HIV care.

*When we meet, we go with the intention of making them feel that we are in their shoes, so that as we chat, they begin to open up. (Lay cadre, Age 25, 2+ years’ experience)*

*Counselling is not only a one‐sided thing; it requires getting each other involved because then you open up to each other. When you just focus on what on what you have to tell them, the person is never open to tell you his problems. It is better to be open, asking them questions and allowing them to also ask you questions. (Nurse, Age 42, 2+ years’ experience)*

In contrast to the recorded counselling session data, the majority of HCWs felt that their counselling sessions usually included tailored action plans for clients. HCWs recognized that developing action plans needed to include active participation from the client; however, only a few lay cadre HCWs acknowledged that they struggled to help clients develop action plans.

*I think it [action plans] doesn't happen often. Most clients have the mentality that the doctor chooses for them. So, the clients say ‘you should tell us’. (Lay cadre, Age 35, 6 months experience)*

*I am confident that with a client I will try to come up with solutions to his situation, but the actual and final determination belongs to the client himself. (Lay cadre, Age 43, 2+ years’ experience)*

HCWs valued being responsive to clients and understood that they sometimes needed to skip some curriculum topics to provide person‐centred counselling based on client needs. This was particularly true for clients who had limited time or interest in the counselling session.

*I used to finish the whole thing [counselling curriculum] every time. But with time I realized that when I reach a place, I should assess the situation of my client. If he is in a hurry, I should learn what his needs are and then plan according to his needs, even if we just discuss four topics I make sure that I understand his views and give him a chance to ask about what we discuss, and maybe go back another time to do other topics. (Nurse, Age 35, 2+ years’ experience)*

### 3.6 Facilitators to quality counselling

HCWs identified specific strategies to facilitate quality counselling. They reported that the job aid facilitated quality counselling because it provided prompts, scenarios and graphics they could use to start conversations with men across various topics.

*[The job aid] guides us and helps the client to be in connection with us. As we go through, the client can see what is there and using pictures the client could easily talk about his stories. It then helps us to see how to enlighten him and how to fix it together. (Nurse, Age 31, 2+ years’ experience)*

*The tool helps us a lot with the examples that are given. Using locally based examples, things that people know, it helps them to understand and connect it before you. It's an advantage that this tool uses local terms to describe medical theories. (Nurse, Age 30, 2+ years’ experience)*

Some HCWs believed that being male helped them motivate, relate and respond to male clients since they could understand what these clients experienced and the pressures they faced. No HCWs believed that their own HIV status (living with HIV or not) affected their ability to counsel, support and build meaningful, lasting relationships with men. Lay cadre HCWs emphasized that men they counselled regularly contacted them for ongoing support even after formal counselling sessions were completed.

*It's because we are also men and we have real experiences and know how to handle that situation when it happened to us. So when we meet a client with that same scenario we can use that experience to help the client. That man‐to‐man arrangement is proving to be good. (Lay cadre, Age 43, 2+ years’ experience)*

A few HCWs identified routine self‐reflection and peer learning as facilitators to improved quality of counselling.

*When I go home, I look at my notes and the questions he asked me and how I responded and think about where I made mistakes. I consult with my colleagues and think about how I can change and approach clients with the same topics but with different ideas. (Lay cadre, Age 43, 2+ years’ experience)*

### 3.7 Barriers to quality counselling

HCWs identified several barriers to quality counselling. First, HCWs believed that clients’ prior experiences with HIV and broader health services could be a barrier because clients held preconceived notions about counselling prior to engaging with this curriculum. A few HCWs from both cadres highlighted that this was particularly challenging for male clients who had previously been on ART for a long period, because they assumed the male‐specific counselling would not be any different from what they had heard in the past and would therefore not meet their needs.

*They just say that they've been hearing about these things for a long time … It was hard to convince the person that there is important information when they are saying they've already heard about it, but still, we try. (Nurse, Age 30, 2+ years’ experience)*

*I have a client who regularly defaults. He told me that all of these things have been told to him before by other people. I explained why I was there and why this was different but it made me feel like I didn't know where to go with the session. (Nurse, Age 37, 2+ years’ experience)*

The length of the counselling curriculum was also identified as a barrier to men's engagement. Counselling sessions usually lasted 40−70 minutes and often conflicted with men's need to earn income. This led to an additional challenge of finding spaces in the community that were fully private for an hour‐long session.

*When you're using a flipchart and asking questions, they lose time [from their business], they feel like they need to do other things and I see that they lose their concentration. (Lay cadre, Age 24, 2+ years’ experience)*

*I've had clients telling me to speak quickly. The [business] environment we were in was not conducive, so I suggested we should find another place and he insisted on talking right there. I told him that if he is busy, we can always reschedule so that he is really free and he told me that if it doesn't happen now, it will be hard to meet again. (Nurse, Age 30, 2+ years’ experience)*

## 4 DISCUSSION

Our results show that both lay cadre and nurses in Malawi can implement a male‐specific, person‐centred counselling curriculum with fidelity and high quality. Counselling sessions mostly used interactive, responsive and respectful strategies that followed person‐centred care best practice and promoted equitable relationships between HCW and client. HCWs took steps to create an open and interactive environment and treated clients with empathy. However, HCWs in our study struggled to help clients identify the next steps to overcome individual barriers and some HCWs did not always fully employ motivational messaging throughout counselling sessions. HCWs were capable of identifying their own successes and challenges with implementation and bought in to a new curriculum. Lay cadre performed as well as nurses across all quality domain measures. HCWs perceived that they excelled at creating interactive sessions that allowed clients to be open but identified the negative impact of prior unsuccessful or unsatisfactory experiences with counselling.

Lay cadre were able to deliver high‐quality, interactive and responsive male‐specific counselling. Our study confirms that lay cadres can implement complex and tailored counselling when provided proper training and job aids, a finding that is important for scalability efforts in resource‐constrained settings like Malawi. Similar findings have been documented in other, similar settings [61, 62]. Lay cadres are an important component of health systems in order to adequately reach ART clients and provide quality services throughout the HIV treatment cascade [63]. Task shifting tailored counselling to lay cadres can reduce the workload of higher‐level cadres and is cost‐effective [64, 65, 66]. Our findings support that with sufficient training, lay cadres can implement tailored counselling for vulnerable populations with fidelity and quality [63, 67].

Several topics central to male‐specific counselling were poorly covered by HCWs: “Self‐Compassion” and “Safe Sex.” “Self‐Compassion” aims to demonstrate that HCWs understand that adherence can be difficult, and that men will not always be “good” clients but the important thing is for them to keep trying and return to care when possible [47]. Men struggling with adherence want to be welcomed back to care and build trust between themselves and HCWs [30]. There are several reasons “Self‐Compassion” may not be adequately included in counselling sessions. The language of this topic may conflict with beliefs some HCWs hold about men as difficult and stubborn clients who are to blame for their own poor use of HIV services [42, 43]. Future implementation of male‐specific counselling should sensitize HCWs to the fact that men engaging with ART are vulnerable to challenges but do care about their health [49, 68, 69] in order to promote the inclusion of new topics like “Self‐Compassion” and sufficiently cover “Safe Sex” to promote holistic sexual health among men.

We found that men were particularly interested in, and had questions on, several key counselling topics: “Attending Health Facilities,” “Side Effects” and “If You Forget ARVs.” Similar themes are frequently cited as key barriers to men's treatment engagement [10, 14] and likely require some of the highest levels of interactivity to meet the needs of men not engaged in care. Future counselling strategies should ensure full discussion of these topics. Men often report feeling unwelcome in health facilities [70, 71, 72] and experience negative interactions with HCWs [8, 9], particularly following treatment interruption [10, 12]. An increased focus on information about “Attending Health Facilities” to address negative facility experiences could improve men's engagement with health services. Interactive counselling on side effects and strategies for dealing with missed medication could fill knowledge gaps and directly address struggles with treatment adherence.

Tying HIV education to the broader goals and values of men is critical to helping men identify how HIV treatment can specifically benefit their lives, and therefore, increases individual buy‐in and commitment to treatment [55, 73–77]. We found that motivating explanations were usually provided “sometimes” not “often.” HCWs need skills to determine when educational topics require additional motivational messaging, and how to tailor messaging to directly tie into individual men's goals and lived experiences. Ongoing professional development may help HCWs navigate such tailored counselling [36].

Counselling sessions rarely included action plans to overcome barriers to care. Understanding what needs to be done to overcome barriers to care and then creating action steps as part of a plan is crucial to improving men's engagement in chronic care services [78, 79, 80]. Programmes in South Africa that include an action‐based component have been shown to help men overcome masculinity‐related barriers to treatment [81]. In our study, HCWs described why action plans were crucial, but struggled to implement the strategy. HCWs who struggled usually attributed this to their ability to elicit active participation from the client. Developing action plans may represent one of the more challenging aspects of person‐centred counselling, requiring counsellors to move beyond pre‐defined curriculums to help clients with on‐the‐fly tailored solutions to challenging problems and unique holistic situations. This skill may be particularly difficult to implement in settings like Malawi where counselling is commonly a lengthy, one‐time event rather than an ongoing process and where lack of resources mean private, one‐on‐one time is also limited. Future trainings on person‐centred counselling should include specific training and job aids on how to quickly identify barriers to care and how to co‐develop action plans, helping HCWs understand how to workshop solutions with clients instead of providing a “laundry list” of potential suggestions. Current systems for those with treatment interruption often focus on the initial re‐initiation [82, 83] which may lead HCWs to focus on the immediate return to care rather than longer‐term adherence. Such adherence requires clients to develop the capacity to overcome barriers on their own, rather than relying on the involvement of HCWs. Programmes should consider how to emphasize and support long‐term engagement for both clients and HCWs.

HCW buy‐in is critical to the success of the new counselling curriculum at scale [84, 85]. Our study suggests that with comprehensive training and gender sensitization, nurse and lay cadre HCWs can buy‐in and successfully implement male‐ and person‐centred counselling. Continued HCW buy‐in may help to promote the feasibility and sustainability of population‐specific counselling. Future programmes must incorporate sensitization and ongoing feedback into routine training and monitoring to sustain HCW buy‐in throughout their career lifespan. HCWs interviewed were clearly able to identify the benefits of male‐specific counselling, felt confident during counselling provision and believed that the curriculum connected well with men as compared to standard of care. Key facilitators to quality implementation were a detailed job aid and time for reflection on counselling sessions. Moving forward, HCWs should be equipped with tools such as a detailed job aid to operationalize skills, and ongoing opportunities for self‐reflection and peer learning.

### 4.1 Strengths and limitations

Our study was strengthened by the use of real‐world counselling sessions that reflect the content and interests of men receiving HIV adherence counselling. HCWs recorded all sessions in which clients consented before we randomly selected a subset for analysis, which means that the analysis accurately reflected HCWs’ skills and limited the possibility that HCWs could selectively implement counselling best practices to elicit a positive review. The study had a few limitations. Counselling sessions were analysed using audio‐recorded sessions only. Videoed or directly observed sessions may provide further insight into HCWs’ ability to provide an empathetic and open environment. HCWs may struggle to be open about challenges in an FGD setting with peers due to social desirability bias. Findings may not be generalizable outside of Malawi. Additional assessments of counselling provision are needed in other settings, especially for vulnerable populations, and with other cadres of HCWs.

## 5 CONCLUSIONS

We found that male HCWs of both lay and nurse cadres were capable of implementing a person‐centred, male‐specific counselling curriculum with a high degree of quality and fidelity. Gender sensitization training plus a detailed job aid helped HCWs to treat male clients empathetically, representing a departure from traditional attitudes towards men in the health system. Programmes should include lay cadres as providers of population‐specific counselling. HCWs need additional support to help clients develop clear action plans to address barriers to care.

## COMPETING INTERESTS

The authors declare no competing interests.

## AUTHORS’ CONTRIBUTIONS

KD is responsible for funding acquisition. KD and TJC conceptualized the study. IR, MM and JAH developed the study protocol and focus group discussion guides and implemented the study. IR, JL, JD and KD developed the analysis plan, codebook and structured memos. IR and JL coded the data. IR analysed the data with support from JL, KD, JAH, KP and EC. IR wrote the first draft and MM, JL, JAH, JD, KP, EC, TJC, MC and KD edited the following drafts. All authors have read and approved the final manuscript.

## FUNDING

This work is supported by the Bill and Melinda Gates Foundation (INV‐001423) and NIMH (R01‐MH122308). KD was supported by Fogarty International (K01‐TW011484‐01).

## Supporting information

APPENDIX A: Male‐Specific Counselling Curriculum

APPENDIX B: Table 3 expanded to show differentiation between nurses and lay cadre HCWs

## Data Availability

The data that support the findings of this study are available from the corresponding author upon reasonable request.
