# Supplementary material for: Implementing a male‐specific ART counselling curriculum: a quality assessment with healthcare workers in Malawi
Source: J Int AIDS Soc. 2024 Jul 22;27(7):e26270. doi: 10.1002/jia2.26270 (PMC11263468; doi:10.1002/jia2.26270)
Supplement: Supplementary file 1 — APPENDIX A: Male‐Specific Counselling Curriculum [file JIA2-27-e26270-s002.pdf]

Men's ART Initiation Counseling Curriculum

Partners in HOPE
"Christ's Love in word and deed"

UCLA

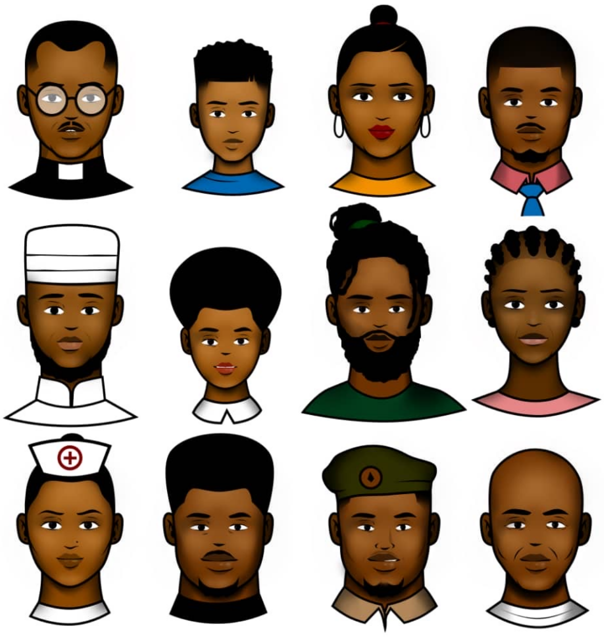

Introduction to HIV

What do you see? Which of these people are HIV-infected?

- Anyone can be HIV-infected. You cannot tell who has HIV by looking at them
- HIV is not a punishment or death sentence – someone living with HIV can live the very same life as someone who is HIV-negative if they take ARVs.

Most important is that YOU are NOT alone.

Almost a million adult Malawians are taking ARVs every day. This means that 1 out of 15 Malawian men are now taking ARVs.

There are many men who have experienced what you are going through right now. And they have overcome. You can too.

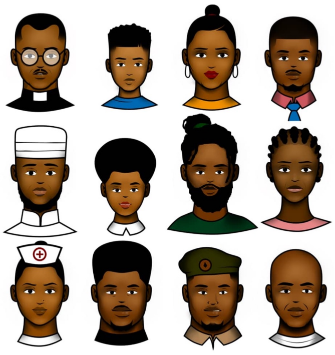

1

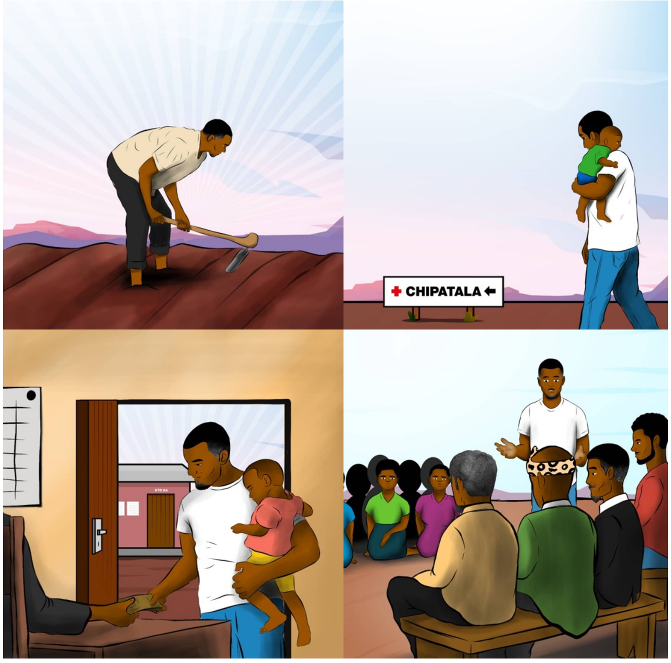

Understanding Men

Our lives are complicated. As men, we have huge responsibilities to provide for ourselves and our families.

What are some of the responsibilities that you personally juggle as a man? (Examples: paying school fees, caring for family).

We have to solve multiple problems every day that need us to be strong and smart – and being healthy makes this possible.

All of us rank our problems and prioritize which ones require immediate attention and which can be solved later. Some problems like HIV may look as if they can be resolved later, but if we do not prioritize them they turn into big problems we cannot easily fix.

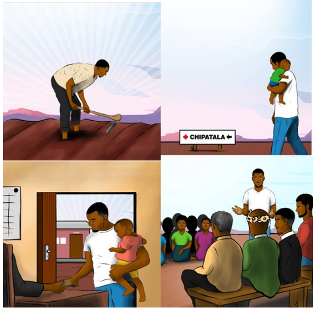

Today I will talk with you to help you decide how to prioritize and
manage HIV alongside all your other responsibilities.

2

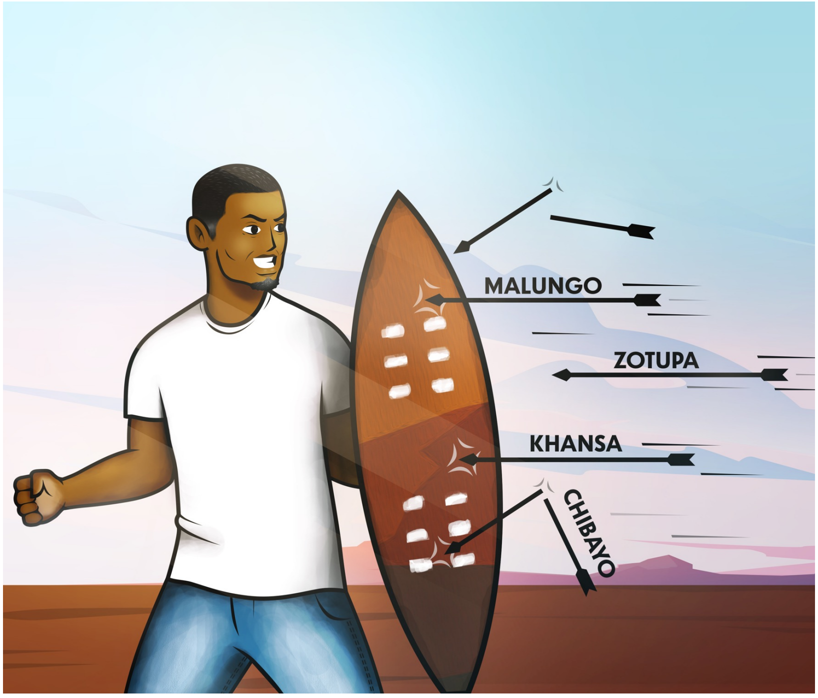

HIV and the Human Body: Immune System

How can you stay healthy?

Look at the picture. The shield is like our immune system. Everyone has an immune system. What does your immune system do?

- It protects you from diseases
- Keeps you healthy
- A healthy body also means a productive and successful man/family/community and country

Your immune system protects you from illness, just like the shield can protect you from arrows. If you get sick, your immune system helps you get better.

As men, our immune system helps us live strong, healthy, productive, and happy life. Only when you are healthy can you realise your dreams of doing business, working in farms, and supporting your families.

What are some of your dreams in your life?

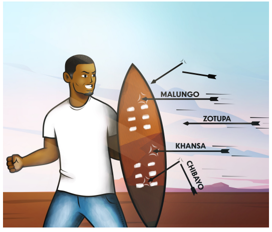

3

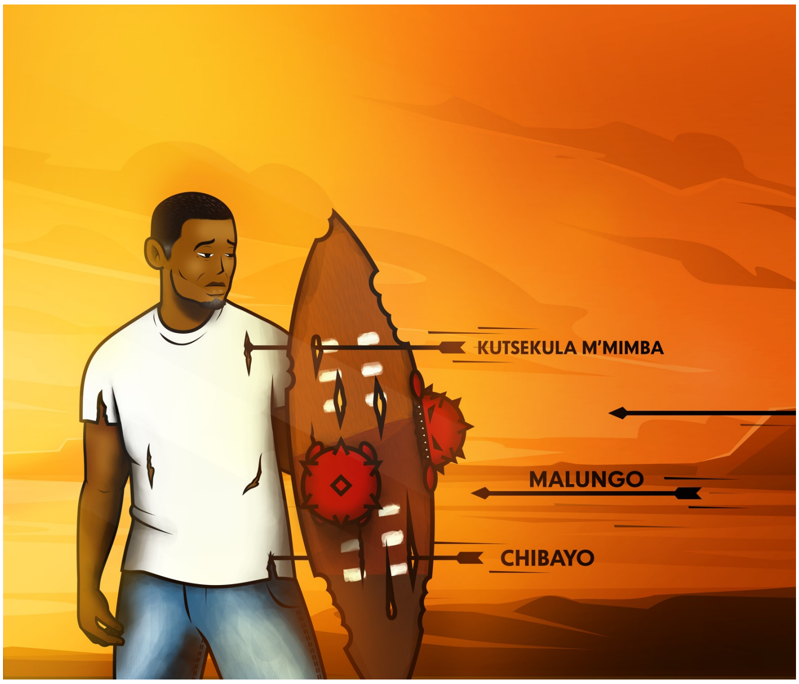

HIV and the Human Body: How does HIV damage your shield?

HIV virus grows and makes more virus every day (multiplies), attacking your shield which is your immune system.

A weak immune system is like a broken shield that lets arrows through. A weak immune system lets more disease and illnesses affect your body.

What happens when your immune system is damaged?

- You will get sicker faster and may feel generally a bit sick
- It can take longer to get better after you have been sick
- Your dreams/goals are negatively affected

Have you or someone close to you ever fallen so sick that you/they could not work? Tell me about it.

- **How did this affect your/their family relations or future plans?**
- **Could going to the doctor earlier helped you/them avoid getting so sick?**

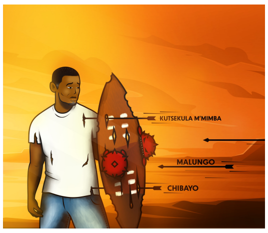

4

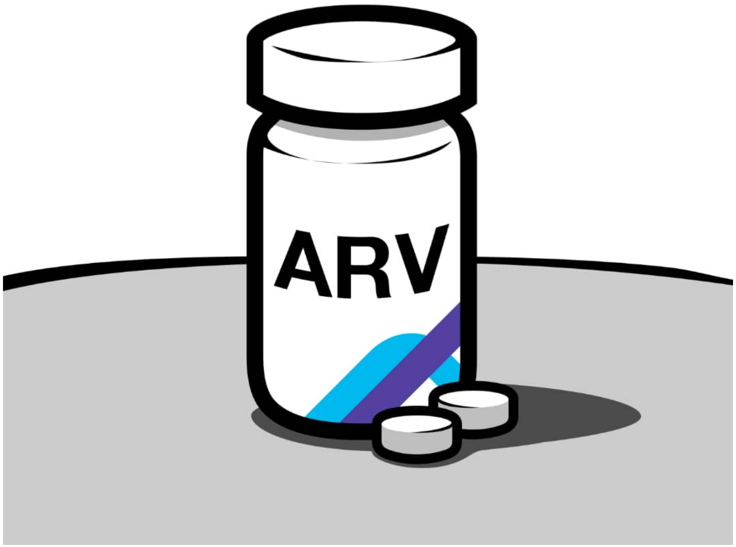

What are ARVs?

ARVs are a medicine made specifically for HIV. We don't have a cure for HIV, but ARVs can let us live a normal life like anyone else. They help control HIV (but not cure it) and rebuild your immune system.

People living with HIV who take ARVs can live a normal life, exactly the same who is HIV-negative.

With ARVs, you will stay looking and feeling healthy, and no one will need to know that you are HIV-positive unless you choose to disclose to them.

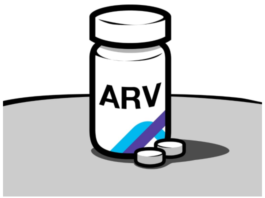

5

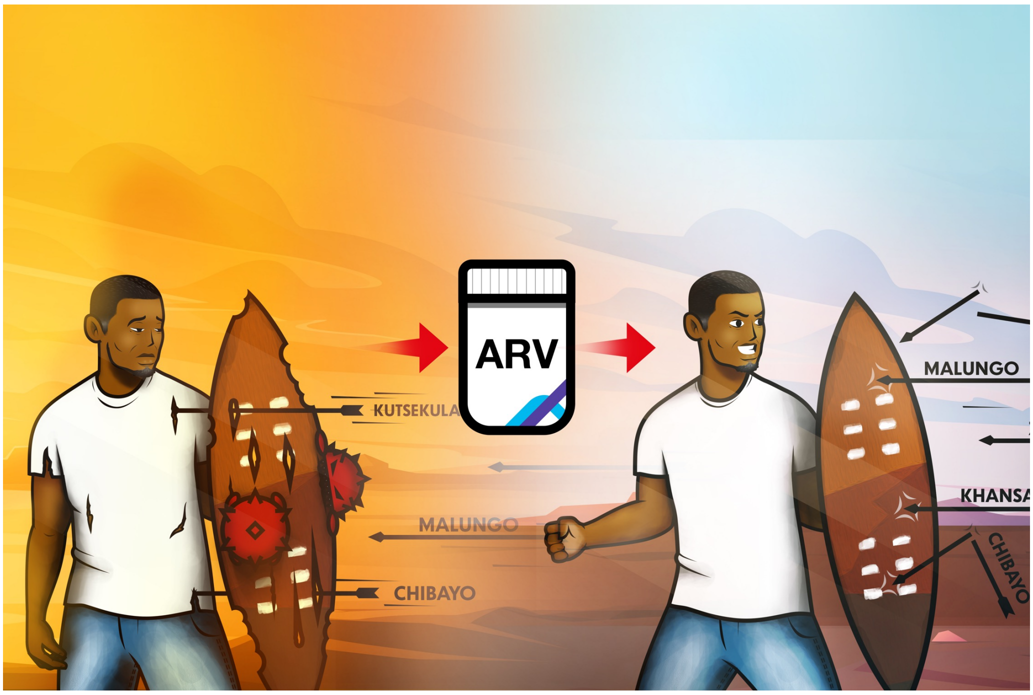

How do ARVs Work?

ARVs make HIV sleep and stop multiplying so that HIV cannot damage your immune system. Your shield becomes strong again so that diseases and illnesses are less likely to affect you.

Less virus in your body means you are less likely to become sick. That way, you can continue to work and live life as usual because the virus is sleeping and cannot work against your body.

Men should be very motivated to take ARVs because it can help ensure that you stay strong, productive and are able to work and stay respected.

How do you think ARVs could help you live your best life?

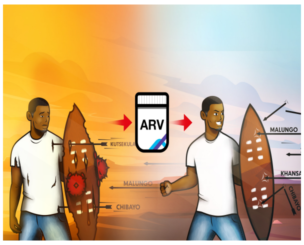

6

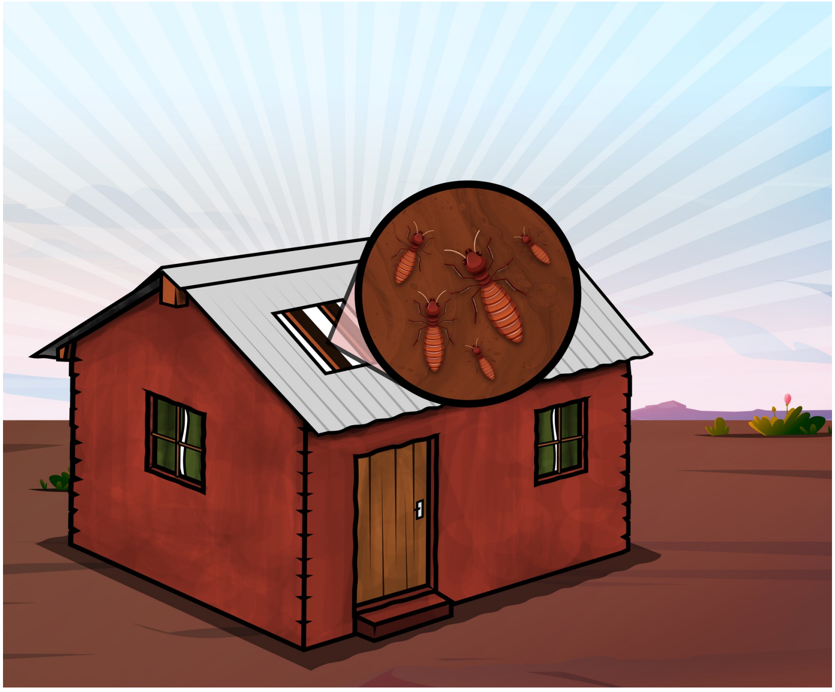

What if you feel healthy now?

Sometimes people feel healthy, even though they have HIV.

How would you describe your health right now? Does your health keep you from doing anything you want to do?

Even if you feel healthy, ARVs are still very important for your body. Even though you can't see HIV or illness on your body, HIV still damages your immune system, making your body more likely to get sick in the future.

Think about a house that is being attacked by termites. Termites work behind the scenes. Sometimes you only know that termites are there after the damage is already done and the house is now ruined. As soon as you find termites you must get rid of them right away to keep your house safe.

Just like termites, HIV works behind the scenes on your body's immune shield. Even if you feel healthy, look healthy, and do not think that HIV is hurting your body inside, it is.

If we don't start ARVs early when we feel healthy, we may end up with more problems. As men, we can't wait until we are sick to take ARVs, because we will loose opportunities to earn money and care for our family.

What are some barriers to starting ARVs when healthy? What could you do to overcome these barriers?

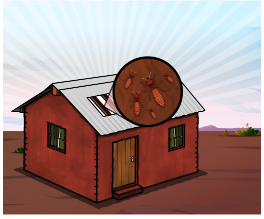

7

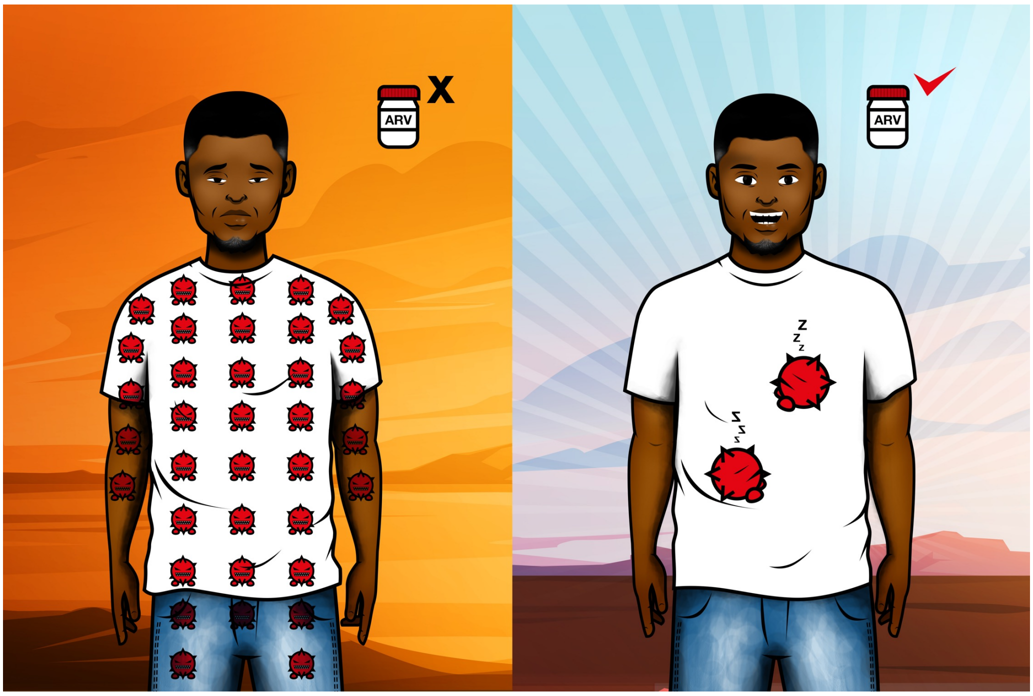

ARVs and Viral Load

HIV viral load tests help us see how much HIV virus is in the body. We take the test 6-months after someone starts ARVs, and every year after that.

When someone is taking ARVs everyday, they will have a low viral load, which means they have less HIV virus in their body and their immune system is strong.

When someone does not take ARVs, they will have a high viral load and their immune system will be weak.

Feeling healthy and a low viral load while on ARVs means the ARVs are working:

- Feeling healthy / low viral load does not mean that HIV is gone
- Keep taking ARVs even if feeling healthy

What do you think men can do to help them take ARVs everyday so they can reach a low viral load?

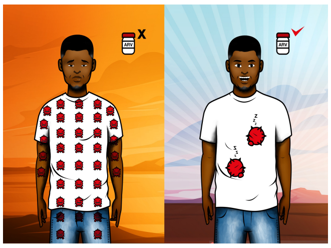

8

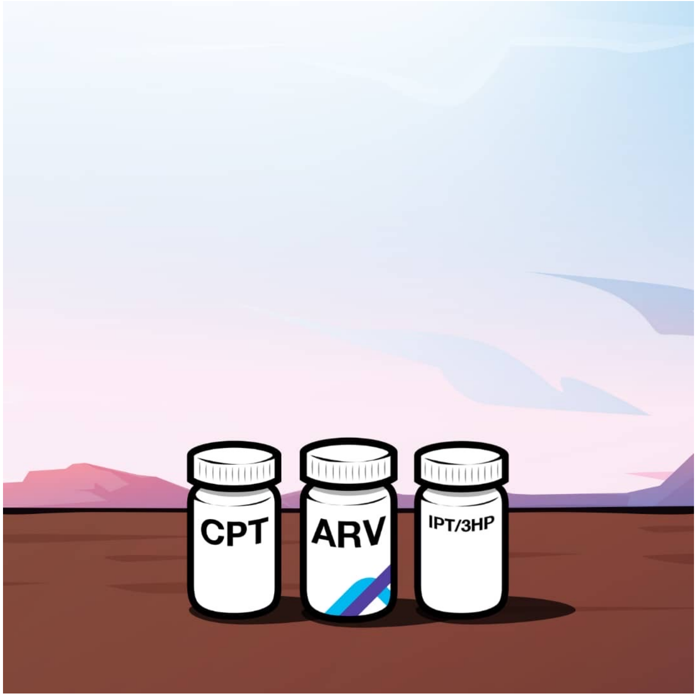

ARVs Do Not Work Alone

ARVs do NOT work alone. Your provider will also give you other medicines that help your shield to stay stronger and healthy.

These are the medicines that will be given to you:

- **CPT or Bactrim:** People with HIV may get illnesses called opportunistic infections. Opportunistic infections include diarrhea, malaria and other bacterial infections and Bactrim helps fight opportunistic infections to help make sure you stay strong and healthy.
- **Tuberculosis (TB) Treatment:** TB is a bacterial infection that effects the lungs and is common among people living with HIV. It is difficult to know if someone who is HIV-positive has been exposed to TB. Because of this, TB drugs are given to all HIV-positive patients as either isoniazid (IPT) or isoniazid-rifapentine (3HP) when they start ARVs. They are also given a vitamin (vitamin B6) to prevent any side effects. TB medications are only taken for 3-6 months and help ensure you remain healthy and can have a productive, normal life.

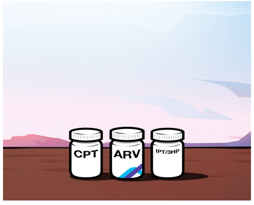

9

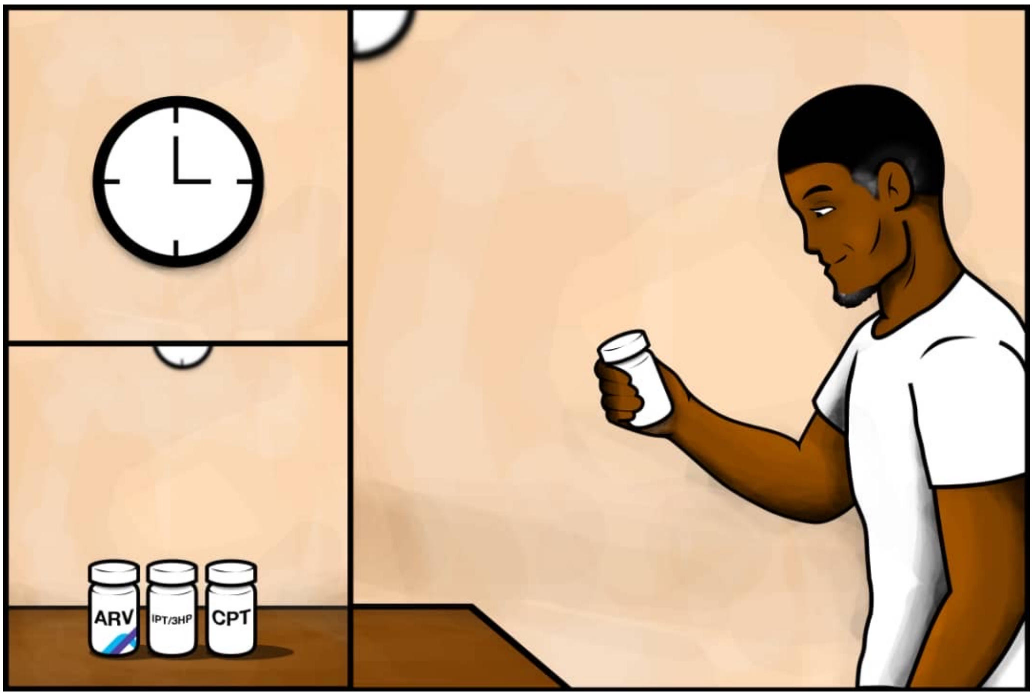

Adherence to ARVs

How do you make sure HIV stays asleep? You take ARVs every day at the same time.

ARVs work best when taken at the same time every day. If you go more than 24-hours without taking ARVs, the ARVs cannot protect your immune system as well until you take ARV again. That's why it's best to take ARVs at the same time every day.

There are three 'rights' for adherence:

- Right time – same time every day
- Right dosage – correct number of pills
- Right drug – know the name of each drug you take every day

What are some methods to help you to take your ARVs at the same time every day?

- Phone alarm
- Someone at home to remind you
- Use sunset/sunrise
- Keep ARV bottle near your bed
- Mark calendar

Lets make a plan. Which of these methods do you think could help you with adherence? Is there anything else you think could help you? – Make a plan -

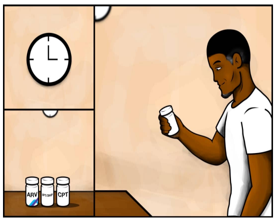

10

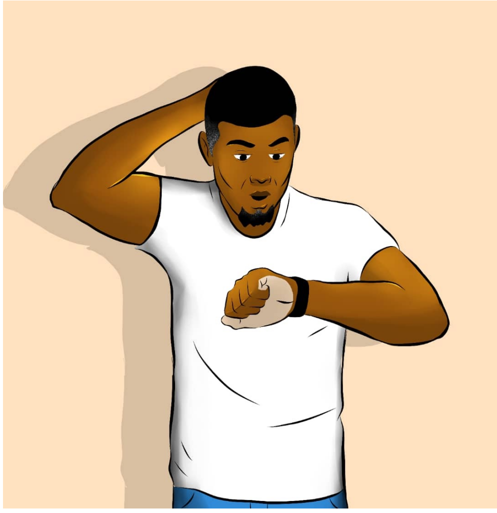

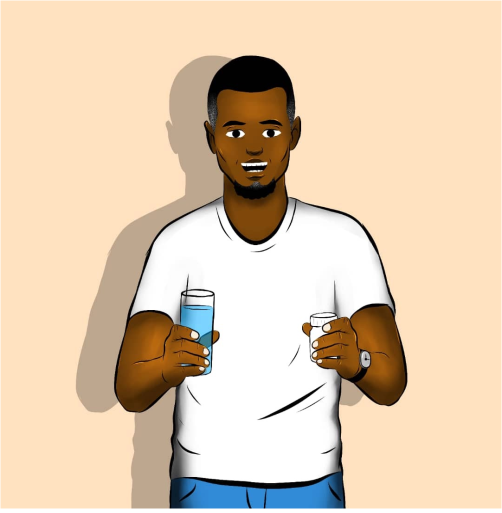

If you forget ARVs

If you forget to take a pill at that exact time just take the pill as soon as you can. If you are a couple of hours late, it's okay! Just return to your normal time the next day. However, never take two pills in one day. If you are more than 12 hours late for taking a pill, it is better to skip the dose and start again the next day.

Do the best that you can, but we are all human. We all make mistakes. Do not be afraid of ARVs because you must take them at the same time very day. We all learn slowly, slowly. With time taking ARVs at the same time every day will become normal, like eating dinner every day.

Are you worried about taking ARVs daily? What are you worried about? What can fix these concerns (either by yourself, with a friend or family member, or with support from a health care worker like me)?

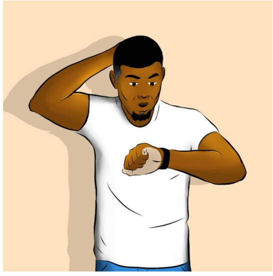

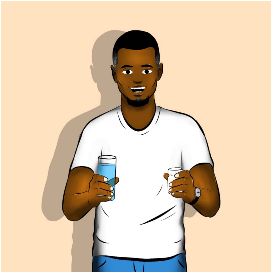

11

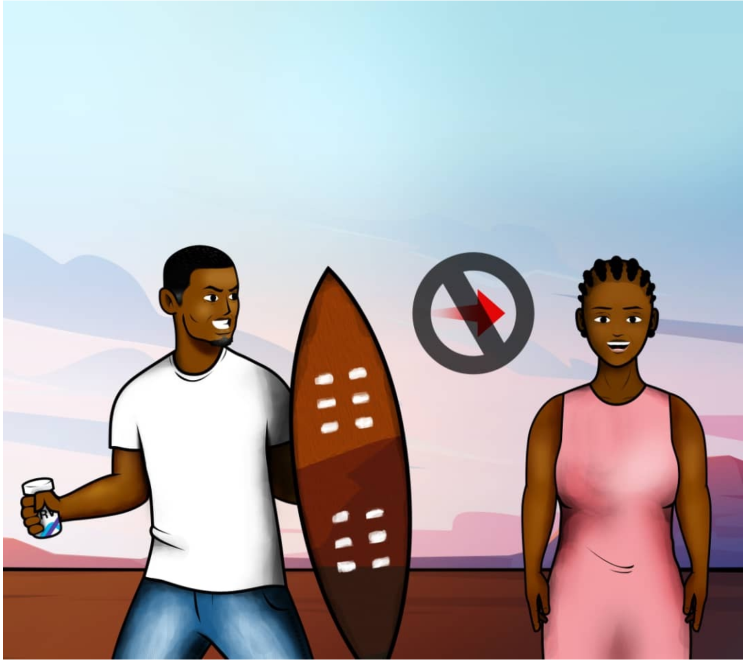

Protecting others from HIV: ARVS

I'd like to get to know a little more about you and your partner.

- **What is your relationship status - are you currently married?**
- **If yes, do you have other sexual partners?**
- **Do you know the HIV status of your sexual partner(s)?**

Did you know that you can prevent passing HIV to a sexual partner by taking your medication? If you take ARVs every day, it is unlikely that HIV will be passed to your partner – your partner can be protected from HIV because you take ARVs.

ARVs help you keep your sexual partners, family and community safe.

By taking ARVs, you are making the best choice for your family. You are protecting your sexual partners health and preparing for the future of your family.

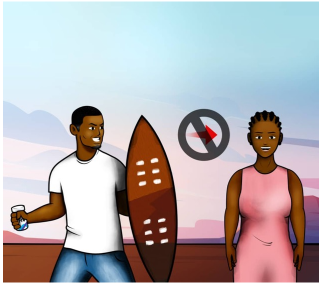

12

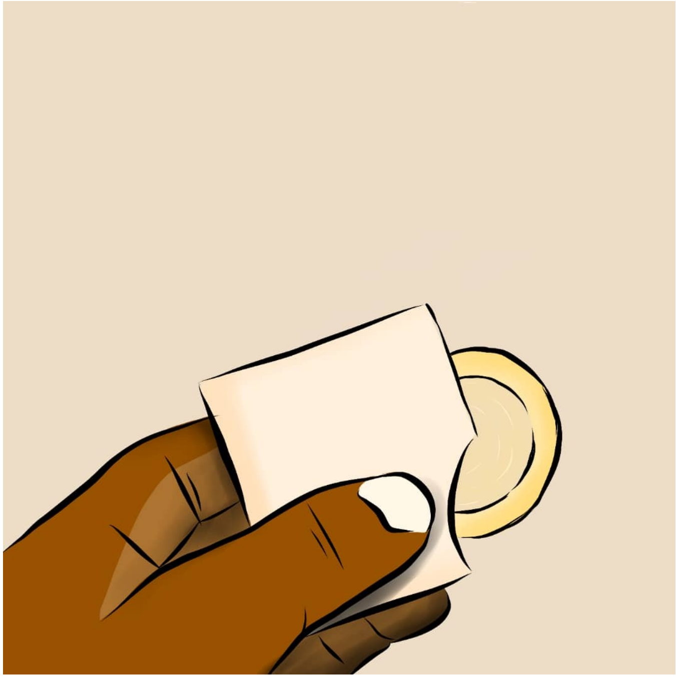

Protecting others: Condoms

If you do not take ARVs, condoms are the only way to protect your partner(s) from HIV.

You should still try to use condoms and try to have fewer sexual partners to protect yourself and your partner from other sexually transmitted infections.

Do you know how to use a male/female condom? *(Explain how to use a condom).* Condoms work only when we use them right. It's not enough to wear a condom – you have to wear it the right way.

Whether you use condoms or not, and no matter the number of sexual partners, ARVs are still good for you and your family and you should start using them right away, even if you do not think you can change your sexual behavior right now.

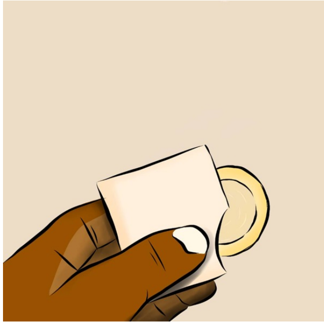

13

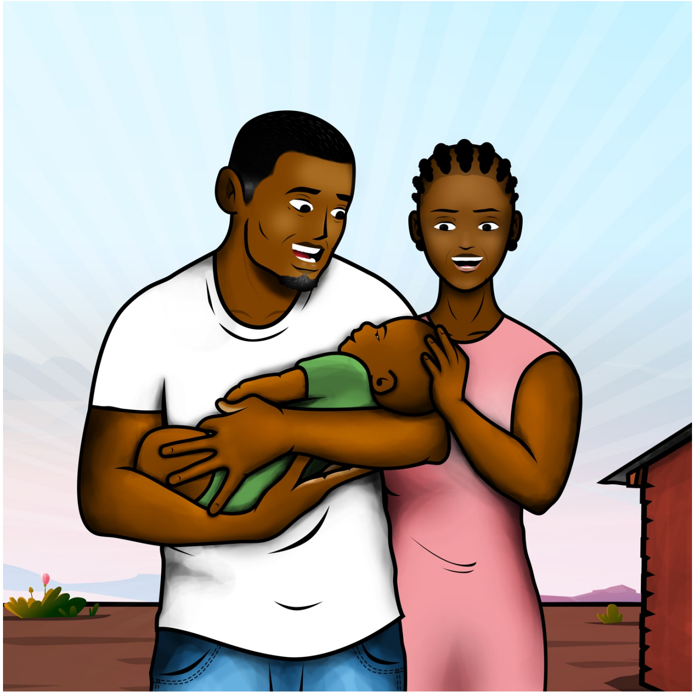

Prevention of Parent to Child Transmission (PPTCT)

Men who are HIV-positive CAN have a child who is HIV-negative? Men are very important in preventing HIV transmission to babies.

You play a critical role and you can have a baby who is HIV negative if you take ARVs consistently every day. If you take ARVs every day, you will have a low viral load and the virus is very unlikely to pass to your partner. If you do not take ARVs, and do not use a condom, you can pass HIV to your partner. Women living with HIV who do not take ARVs everyday can then pass HIV to her baby, either while she is pregnant, or during breastfeeding.

Are you interested in having children sometime in the future?
How can ARVs address some of your concerns with having a child?

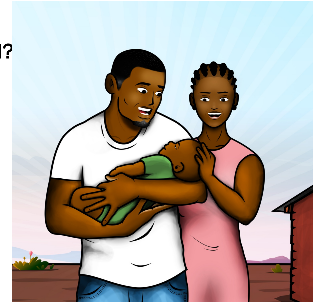

14

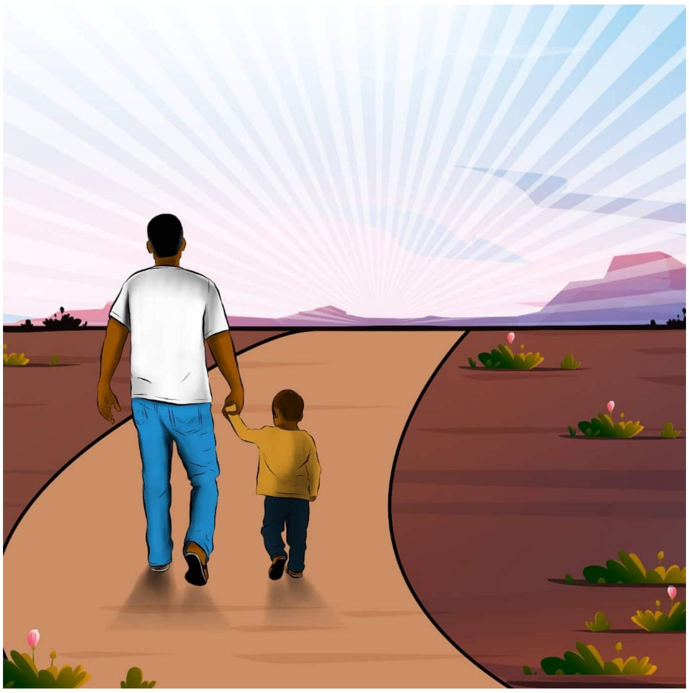

Prevention of Parent to Child Transmission (PPTCT)

As men, how can you help to make sure that HIV is not passed to your baby?

- Use a condom every time you have sex
- Take your ARVs every day
- Encourage and support your partner to attend ANC and test for HIV regularly
- If your partner is HIV positive, encourage her to take ARVs daily, especially in her pregnancy and while she is breastfeeding

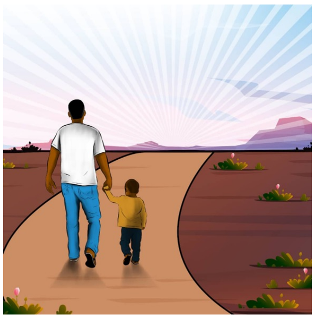

15

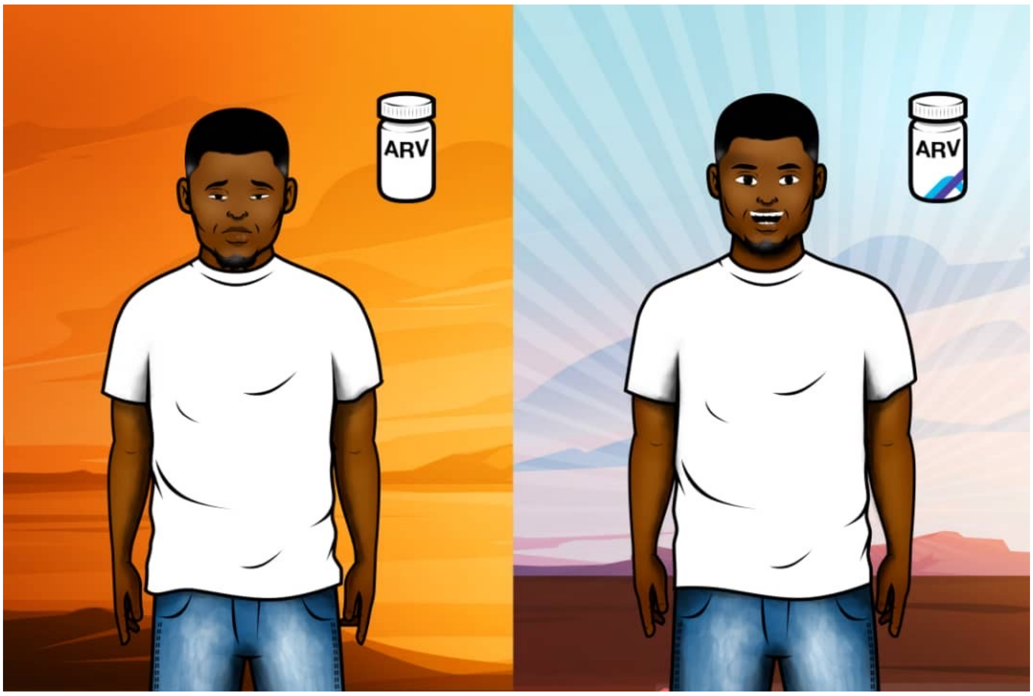

Side Effects: New vs Old ARV Regimen

Have you heard of or experienced negative side effects from old ARV regimens? What is it? What are your fears/concerns with side effects from ARV?

Some men do not start taking ARVs because they heard about side effects. The good news is that the new ARVs that were introduced in Malawi in 2019 have VERY few side effects, if any, and they do not last long.

Most people do not have any side effects at all with the new ARVs. Occasionally people can feel dizzy or have some nausea, diarrhea, or stomach pain in the first few days after starting ARVs. These symptoms usually go away within 2-3 days. Some people also gain weight. It is important to exercise and eat a healthy diet in order to be as healthy as possible and fulfill your dreams.

You should discuss any concerns about side effects with your provider. It is very important that you don't stop taking ARVs unless your provider tells you to.

Taking ARVs for life will not poison you. It is not possible for your body to become 'saturated'. ARVs have been made to work WITH you, not against you.

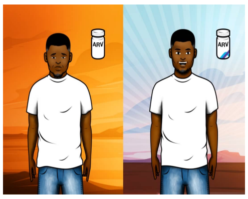

16

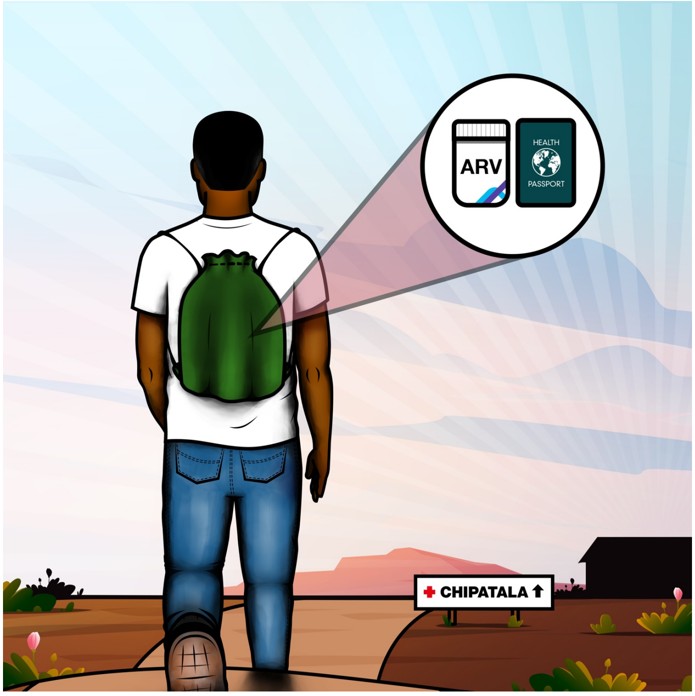

Visiting a Health Facility

ARV clinics may feel uncomfortable. But health care workers like me are there to help you and make you feel comfortable. Most ART clinics open from around 8:00-1:00, depending on what other work providers are doing and number of patients. If you arrive early, you can hear a health talk.

What should you bring to your ARV appointments?

- Health passport book
- ARV, TB, and CPT bottles.

What do you do if you are uncomfortable with the ART clinic or you feel that someone treats you badly? You have options.

- Report to the Health Advisory Committee (HAC) for that facility - contact number posted at facility.
- Talk to another provider or worker who should be able to help you
- Transfer to another facility you think may be more friendly / where you feel more comfortable
- I can help you navigate a facility to make sure you are comfortable and everyone treats you well.

Have you had bad experiences at a health facility/ ART clinic? What happened? What concerns do you have about going to the facility again? How do you think you can avoid bad experiences again or address your concerns?

We know providers can be rude sometimes and sometimes health services are poor. Your health and your family is more important than your experiences at a facility. Even if you do not like going to the facility, still you must find a way to take ARVs so your body can stay healthy and your family protected.

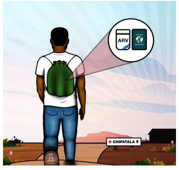

17

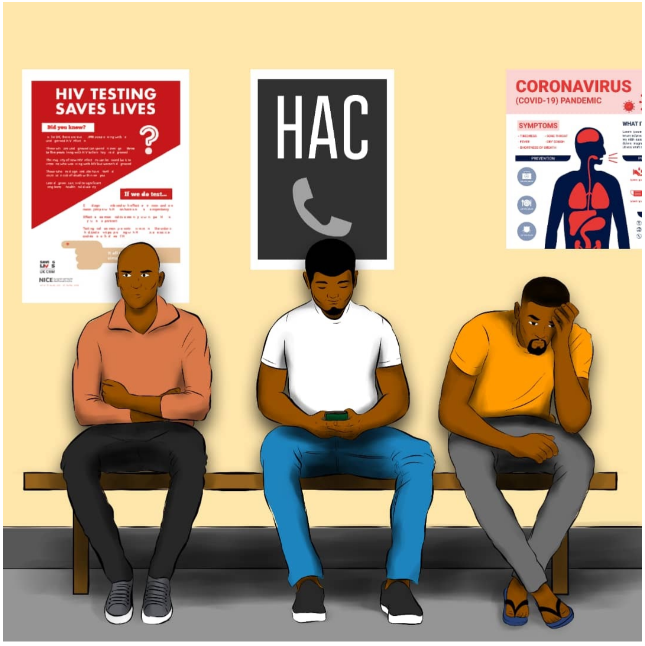

Experiences with Health Facilities

Do you have any past experiences with the ART clinic?

What was it like?

How did you feel when you were there?

How were your interactions with providers?

What do you do if you are uncomfortable with the ART clinic or you feel that someone treats you badly? You have options.

- You can report this to the Health Advisory Committee (HAC) for that facility - each facility should have a HAC (it is like a PTA for schools, but for health facilities) and should have the contact number for the HAC posted on the facility's wall.
- You can talk to another provider, Patient Supporter, or HSA who should be able to help you
- You can also transfer to another facility that you think may be more friendly / where you would feel more comfortable

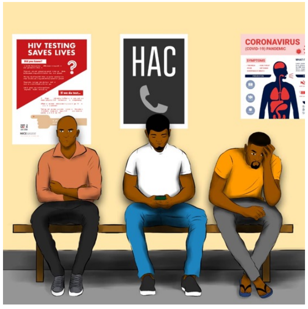

18

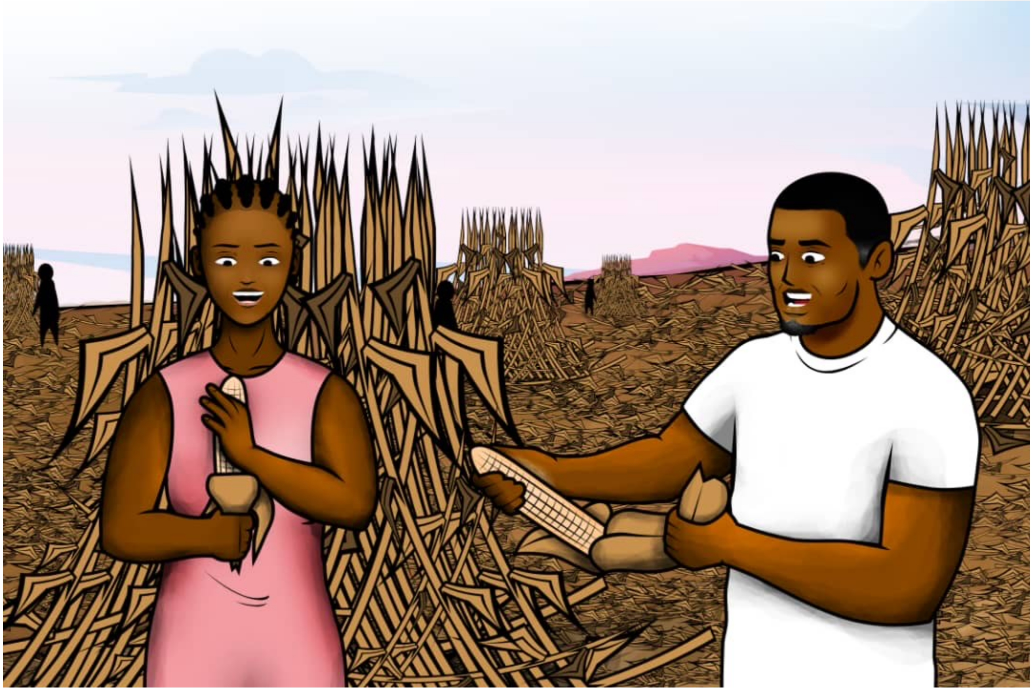

Guardian Support

We all need support in life's journey. It will help you if you have support from a friend or family member who can help you get ARVs and take them every day. For most men, having a guardian they can trust with their HIV status and ARVs is very important to their treatment and to their success in life.

A guardian can:
- Encourage you that you look strong and healthy
- Remind you to take your pills every day
- Support you when you feel scared or nervous about unwanted disclosure
- Help you get to the facility and deal with any challenges at the facility
- Collect your ARV's if you cannot do so yourself / so you don't have to go every time
- Help cover for you at work or with friends when you go to the facility (to avoid unwanted disclosure)

Thinking about these different kinds of support, what do you think would be most helpful for you?

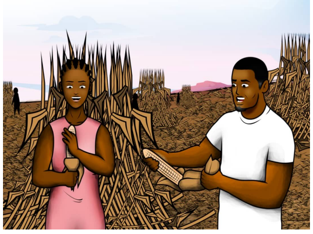

19

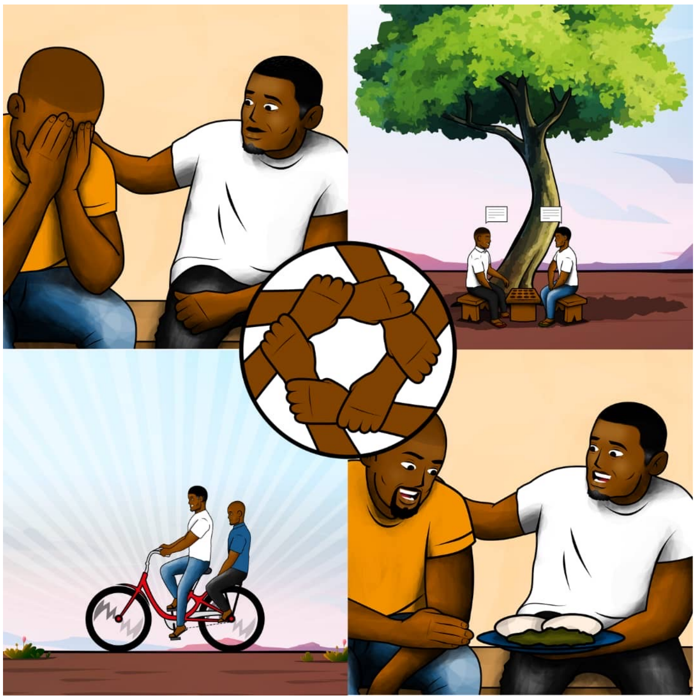

Male Guardian Support

Would you consider talking with men in your life? (e.g., brothers, brothers in law, friends, peers, pastor etc.) Men find it helpful to disclose to other men because:

- Male guardians share similar responsibilities and dreams and can easily relate with what you are going through
- Male guardians can be helpful with transport and escort you to the facility and elsewhere
- Male guardians can help with resources such as food and other needs

Remember it is best to have at least one person who is willing to visit the facility for you so you do not have to go all the time.

Do you have someone like this that you can trust? It is often best to have a few people you trust so you have more support. Who would be a perfect guardian(s)?

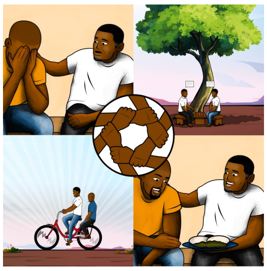

20

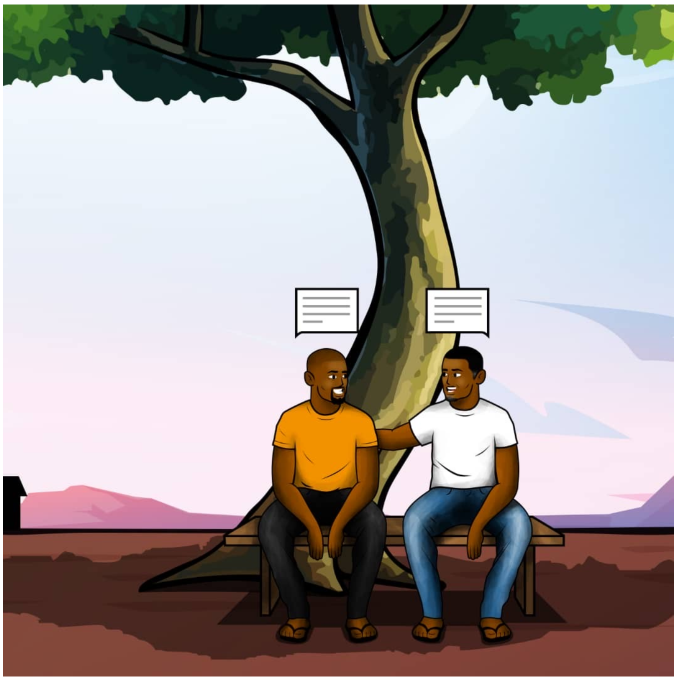

Disclosure

Have you disclosed your HIV-status to anyone? Who? If you have not disclosed, what is stopping you from telling someone your status? Who could you tell?

Disclosing your status not only helps you build a support network to go to the health facility or help you remember to take ARVs, it also helps you feel comfortable and accepted by those close to you. **Disclosure can strengthen your relationships because you can be honest.**

Disclosure works best when you feel comfortable, ready to disclose, and do not feel pressured. It's OK if it takes some time to become comfortable telling someone your status.

When you decide to disclose, think about when, where and how you will tell the person.
Plan for a good place and time to have the conversation, for example when their children are asleep and no one else will hear.

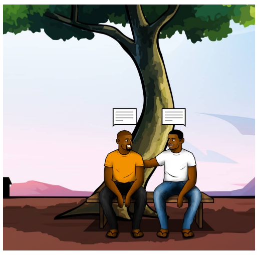

21

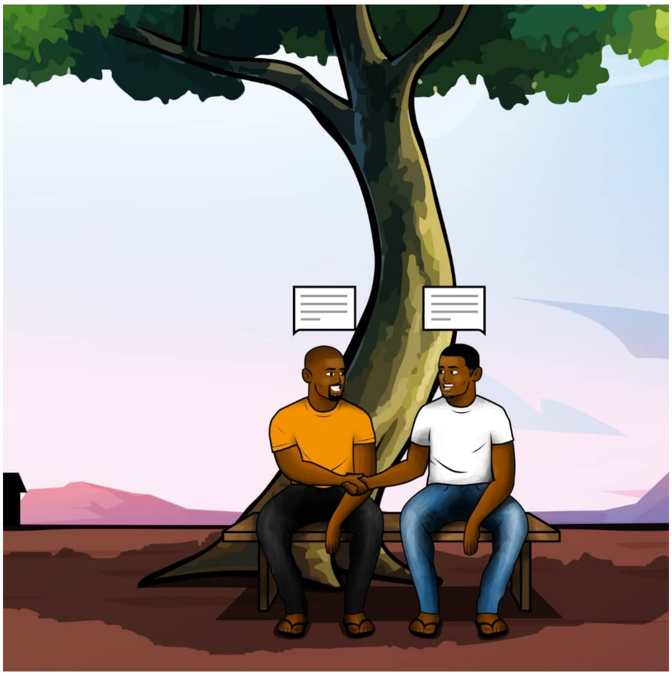

Disclosure

How do you disclose?

- Think about a beginning script
  - Maybe you can say 'I wanted to talk to you about something because I know you can help and support me', or 'because I trust you and value your friendship/relationship'.
- Be prepared that they will have questions for you to answer.
- Remember that they want to understand so share as much as you can.
- Discuss how they can best support you and when.

If you disclose to your sexual partner, try to imagine how you would want to be told about their HIV status. Reassure them that you can stay healthy and can take treatment to help protect them and others from HIV.

Can you think of anyone in your life you would like to disclose to?
What are you most afraid of with disclosure?
What do you think could be done to help you not be afraid?

We are here to support you during your disclosure process. I can also go with you to disclose or help you practice exactly what you will say.

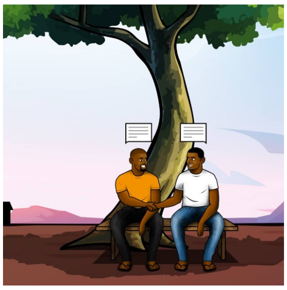

22

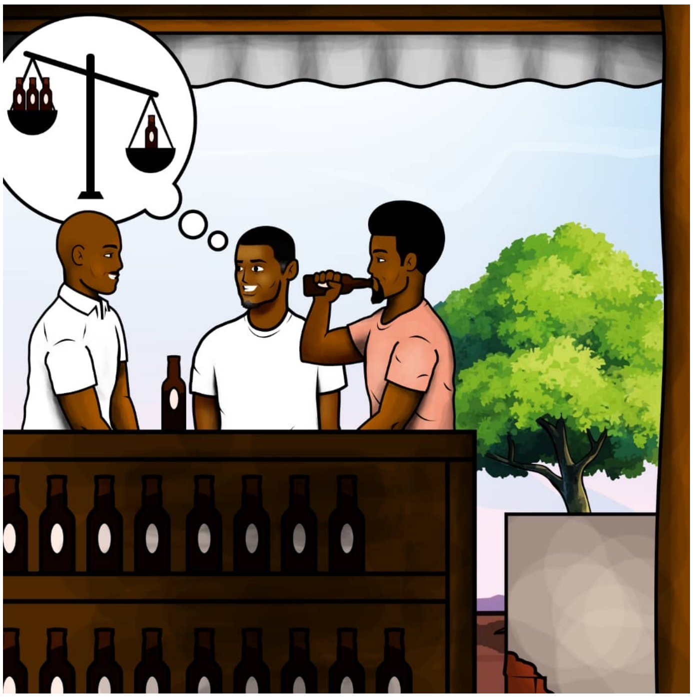

Alcohol

Do you drink alcohol? Regularly or occasionally? What have you heard about ART and drinking alcohol?

Some men do not start ARVs because they think they can not stop drinking beer. Others feel that they may not be able to take ARVs regularly and on time if they drink alcohol.

It is true that managing your drinking may help you take your ARVs more consistently because people can forget to take doses when they are drinking or hung over.

Drinking less is better for your health, but this does not mean that you have to stop drinking completely in order to take ARVs. **You can still drink a couple beers a week while on ARVs, as long as you remember to take your ARVs at the same time every day.**

If you cannot stop drinking alcohol, you can and should still start ARVs.

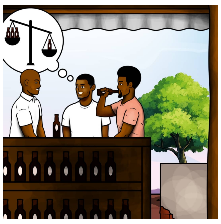

23

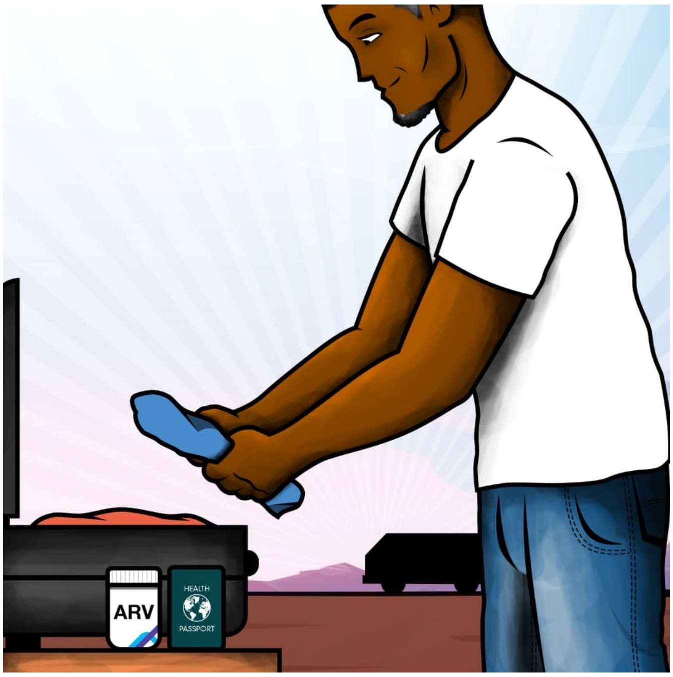

Traveling and Being Away From Home

As men we have huge responsibilities and may have to spend nights away from home for extended periods of time. We may spend some days or weeks away from home for business or family.

Pack all of your medication and your health passport book when you travel.

If you forgot your ARVs or ran out of ARVs when traveling, you can get an **emergency supply** from the nearest ARV clinic. Follow these steps:

- Go to the nearest health facility that has an ARV clinic
- Bring your health passport, show your passport to the provider and ask for emergency ARVs from the ARV clinic
  - If you forgot your health passport, you should still go to a facility and ask for an emergency supply – tell them how long you have been on ARVs and the type of drug you take.

Do you often spend nights away from home? Are you concerned that travel will make it difficult to take ARVs? Why? What do you think could be done to make ARVs easier while traveling, or once you
return back home?

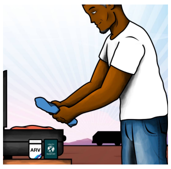

24

If you miss your facility ARV appointment it's OK. We are all busy. Come back to the facility as soon as you can on a regular ARV clinic day and we will be very happy to help you get back on a regular ARV schedule.

[illegible]

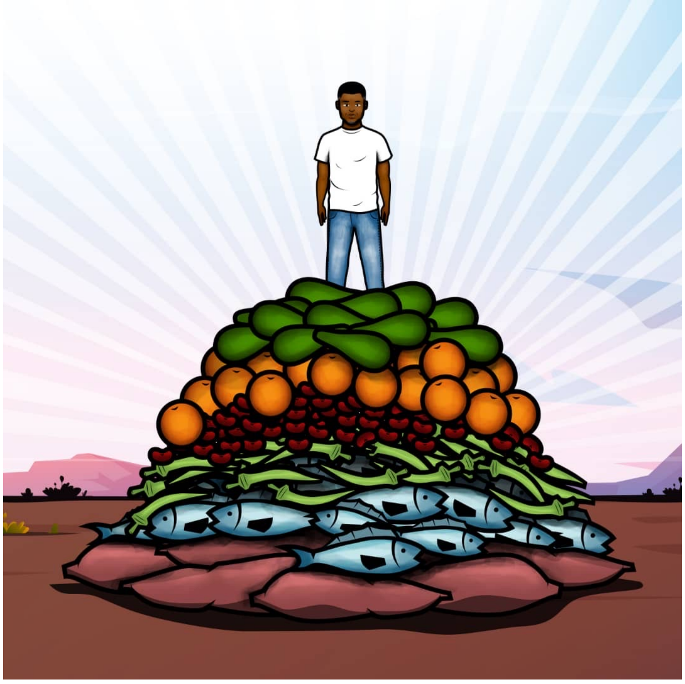

Eating Healthy Foods

Eating healthy foods can help you stay extra healthy and you should try to eat healthy foods. **But if you don't have food, or healthy food, this should NOT stop you from starting or continuing to take ARVs.**

Some men decide not to start ART because they cannot keep eating a good diet which they may think is too expensive. But there many local foods that are healthy and recommended. And the new ARVs are so good that the food you eat is not so important.

Good foods include:
Staple foods include foods high in carbohydrates, like whole grain maize flour (mgaiwa), porridge, Irish potatoes, cassava, sweet potato, rice, green bananas, millet, sorghum, yams, coco, and wheat

Animal foods group includes eggs, meat, milk products, fish, ngumbi, mbewa, etc
Vegetable group includes dark leafy vegetables (mpiru, bonongwe, chisoso, therere, pumpkin leaves, kholowa, khwanya, chigwada, etc.)

Legumes group includes ground nuts, soya beans, beans, peas, cowpeas, ground beans (nzama), and pigeon peas.

Fruit group includes oranges, lemons, and tangerines, bananas, pineapple, pawpaw, mangoes, masau, bwemba, malambe, masuku, peaches, apples, guava, water melon and many others

Fats group includes oil seeds (soybeans, groundnuts, and sunflower seed), avocado (pear), cooking oil, milk and milk products such as butter, margarine, yoghourt, meat, fish, and poultry

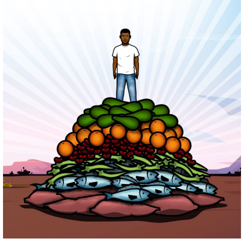

25

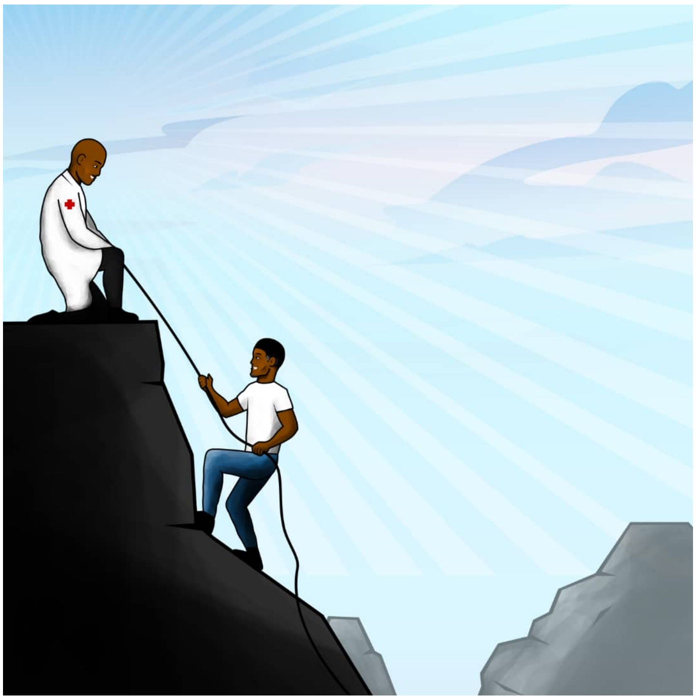

Little By Little You Can Succeed

You CAN take ARVs and live a healthy, normal, and productive life. Some men feel that it is a burden to take ARVs for life. **But many men have realized that reaching their dreams and being alive for their family matters more than the burden of taking ARVs.**

As men, we may struggle to take ARVs well for the rest of our life. But the struggle is okay as long as we keep trying – we grow slowly. We know that men love their families, their friends, and work hard to provide. They may fail at times but they always find a way to try again. **Therefore, you must give yourself grace and not be afraid of potentially failing to take ARVs well.** Keep trying each and every day and prioritize HIV alongside all your other responsibilities sso HIV does not turn into a big problem that keeps you from working and preparing for your future.

With support from the people around us and from the facility, little by little you will succeed. Think about a grasshopper, how with just one hop he moves a far distance. Tsokonombwe anatha mtunda ndi kudumpha.

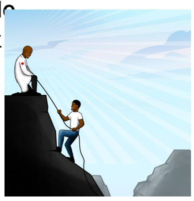

Do you have any concerns about taking ARVs for life? What can you tell yourself to help overcome these fears?

26

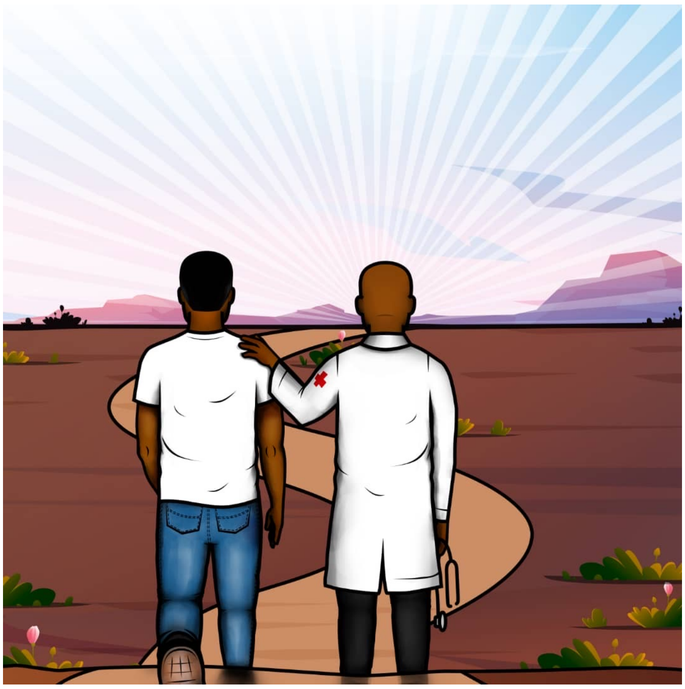

Conclusion

You are not in this alone – we can face HIV together! I am here to support you.

Do you have anything that you are worried about or want to know more about?
Everyone has different worries about starting ARVs, what can help you start ARV?

Many times we are worried that HIV will stop us from reaching our dreams or providing for our families. But by (re)starting ARVs as soon as possible HIV goes to sleep in our bodies and we can live our lives just as if we were HIV-negative.

Taking ARVs can become simple and HIV should not be a reason to put your dreams on hold, or not provide for your family.

Health care workers like me are here to help you and can:

- Go with you to the facility to help you go through the ARV clinic
- Help you make a plan to take ARVs every day
- Help you disclose to your family or friends
- Help you make a plan on how to live a normal life when taking ARVs
- Refer you to resources in the community

Is there anything else I can help you with?
Do you have any other questions?

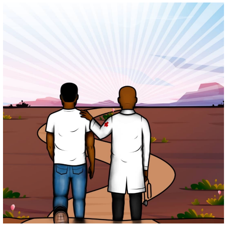

27
