# Supplementary material for: Implementing a male‐specific ART counselling curriculum: a quality assessment with healthcare workers in Malawi
Source: J Int AIDS Soc. 2024 Jul 22;27(7):e26270. doi: 10.1002/jia2.26270 (PMC11263468; doi:10.1002/jia2.26270)
Supplement: Supplementary file 2 — APPENDIX B: Table 3 expanded to show differentiation between nurses and lay cadre HCWs [file JIA2-27-e26270-s001.pdf]

APPENDIX B: Table 3 expanded to show differentiation between nurses and lay cadre HCWs

	Respect		Open		Respond		Inform		Act		Motivate	
n=50<br>Nurse, n=22<br>Lay Cadre, n=28	Accepting/Non-judgmental Language Used Toward Client n (%)		Open-Ended Questions Asked by Counsellor n (%)		Follow-Up Engagement Made by Counsellor n (%)		Client Questions Answered in Detail by Counsellor n (%)		Action Plan Developed n (%)		Motivational Explanations Provided by Counsellor n (%)	
	Nurse	Lay Cadre	Nurse	Lay Cadre	Nurse	Lay Cadre	Nurse	Lay Cadre	Nurse	Lay Cadre	Nurse	Lay Cadre
**Frequent**	22 (100)	28 (100)	18 (82)	18 (100)	11 (50)	14 (50)	11 (50)	16 (57)	3 (14)	6 (21)	3 (14)	3 (11)
**Sometimes**	0 (0)	0 (0)	4 (18)	0 (0)	10 (45)	12 (43)	0 (0)	0 (0)	N/A	N/A	15 (64)	21 (75)
**Never**	0 (0)	0 (0)	0 (0)	0 (0)	1 (5)	2 (7)	1 (5)	0 (0)	19 (86)	22 (79)	4 (18)	4 (14)
**N/A**	N/A	N/A	N/A	N/A	0 (0)	0 (0)	10 (45)	12 (43)	N/A	N/A	N/A	N/A
